# Evaluation of developmental toxicity of chlorpyrifos through new approach methodologies: a systematic review

**DOI:** 10.1007/s00204-024-03945-6

**Published:** 2025-01-27

**Authors:** L. Coppola, G. Lori, S. Tait, M. A. Sogorb, C. Estevan

**Affiliations:** 1 Center for Gender-Specific Medicine, Istituto Superiore di Sanità, Rome, Italy; 2 Bioengineering Institute, Miguel Hernández de Elche University, Elche, Spain; 3 Applied Biology Department, Miguel Hernández de Elche University, Elche, Spain

**Keywords:** Chlorpyrifos, Developmental toxicity, New approach methodologies, NAMs, Systematic review

## Abstract

Chlorpyrifos (CPF) is an organophosphorus pesticide of concern because many in vivo animal studies have demonstrated developmental toxicity exerted by this substance; however, despite its widespread use, evidence from epidemiological studies is still limited. In this study, we have collected all the information generated in the twenty-first century on the developmental toxicity of CPF using new approach methodologies. We have critically evaluated and integrated information coming from 70 papers considering human, rodent, avian and fish models. The comparison of the collected evidence with available adverse outcome pathways allows us to conclude that adverse outcomes observed in animals, such as memory and learning impairments as well as reduction in cognitive function, could involve several mechanisms of action including inhibition of acetylcholinesterase, overactivation of glutamate receptors and activation of mitogen-activated protein kinase, extracellular signal-regulated kinase 1/2, followed by both disruption of neurotransmitter release and increase in oxidative stress and apoptosis.

**Supplementary Information:**

The online version contains supplementary material available at 10.1007/s00204-024-03945-6.

## Introduction

**Fig. 1:**
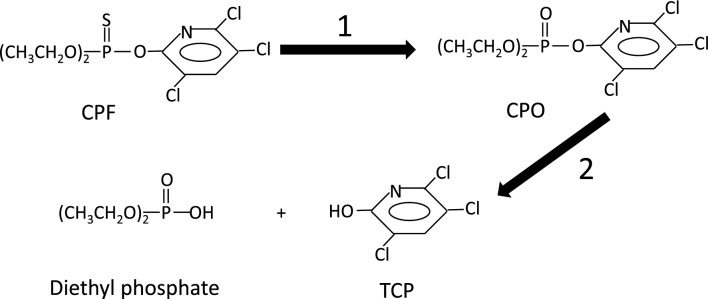
Biotransformation pathways of chlorpyrifos. Reaction 1 is an oxidative desulphuration leading to toxic activation. Reaction 2 is a hydrolysis leading to detoxification. *CPF* Chlorpyrifos. *CPO* chlorpyrifos-oxon. *TCP* 3,5,6-trichloro-2-pyridinol

Organophosphorus pesticides are widely used chemical pesticides. It is well known that one of the major toxic effects of these compounds is the inhibition, by irreversible phosphorylation, of esterases in the central nervous system (World Health Organization (WHO) 1986). In particular, inhibition of acetylcholinesterase (AChE) causes an acute cholinergic syndrome, which is the main molecular mechanism of insecticide action. Chlorpyrifos (CPF) (O, O′-diethyl-O-3,5,6-trichloro-2-pyridyl phosphorothionate) is one of the most widely used agricultural insecticides. Like most organophosphorus pesticides, CPF is manufactured and applied in the ‘thio’ form (Fig. 1), which is stable and with poor AChE inhibitory properties (WHO 1986). However, following ingestion, CPF is bioactivated (through an oxidative desulphuration) to chlorpyrifos-oxon (CPO) (Fig. 1), which is the active metabolite of CPF with high capability of AChE and other esterase inhibition (WHO 1986). CPO is finally detoxified by a hydrolysis reaction yielding diethylphosphate (a substance with no ability to phosphorylate esterases) and 3,5,6-trichloro-2-pyridinol (TCP), which is commonly used as a biomarker of exposure to CPF in urine samples.

CPF is included in the Agency for Toxic Substances and Disease Registry 2022 Substance Priority List in the position 64 out of 275 (https://www.atsdr.cdc.gov/spl/index.html#2022spl). In 2019, EU did not renew the approval for CPF and CPF-methyl, on the basis of a European Food Safety Agency (EFSA) statement declaring that no safe exposure level could be established for these compounds (EFSA 2019), which means that CPF poses a significant potential threat to human health. This decision is based on the growing body of evidence indicating that CPF may be involved in genotoxicity and neurodevelopmental toxicity.

**Fig. 2:**
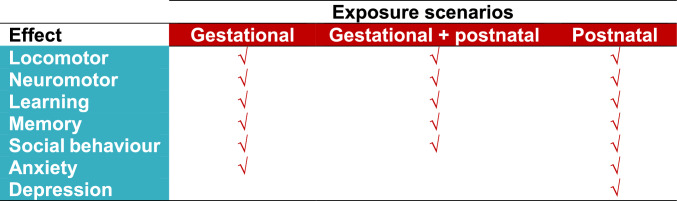
CPF exposure scenarios and neurobehavioural effects in studies with animals. Data taken from Silva (2020)

CPF can cross the placenta, thus representing a harm for the developing foetus. Indeed, the evidence based on animal studies suggesting that CPF is a neurodevelopmental toxicant is well established, and it is well demonstrated that CPF can cause, among other effects, long-term spatial learning and memory impairments (López-Granero et al. 2016; Terry Jr. et al. 2012; Guardia-Escote 2018), anxiety and distress (Ribeiro et al. 2022), puppy ultrasonic vocalisations (Berg et al. 2020) and social changes (Biosca-Brull et al. 2022; Venerosi et al. 2015) also with sex-specific outcomes. Silva (2020) reviewed the effects of low non-cholinergic doses of CPF on rodent neurobehaviour and concluded that in all three possible exposure scenarios (gestational, gestational + postnatal and postnatal), CPF elicits locomotor and neuromotor changes (with no consistency in the reports as to whether the effect is an increase or decrease), reduction in cognitive performance (learning and memory) and increase in social behaviour (Fig. 2). The prenatal and postnatal exposures induce a reduction in anxiety, while the postnatal exposure induces an increase in depressive behaviour (Fig. 2) (Silva 2020).

A review of epidemiological studies analysed developmental and neurobehavioural outcomes in association to CPF exposure in three different cohorts with sample sizes ranging from 254 to 828 individuals, concluding that there is insufficient evidence to link developmental exposure to CPF and adverse neurobehavioural effects in infants and children (Li et al. 2012). In a follow-up study considering the same three cohorts and adding a fourth, the authors reported no consistent findings and no strong association between biomarkers of CPF exposure and foetal growth (Mink et al. 2012).

He and co-workers (2022) reviewed the experimental evidence for a possible association between maternal exposure to various pesticides during pregnancy and the subsequent development of autism spectrum disorders. For the specific case of CPF, three different case–control studies involving 2961, 296 and 486 pregnant women were considered. The odds ratios were 1.1, 2.3, and 2.0–3.3, respectively, depending on the trimester of pregnancy. In another case–control study, the association between autism spectrum disorder and exposure to the pesticide diazinon (a structural analogue of CPF) showed an odds ratio of 1.1 (He et al. 2022). Therefore, the reported evidence supports an increased risk to develop autism spectrum disorders following CPF prenatal exposure, but more studies are needed to substantiate this finding.

Beside exerting neurodevelopmental adverse effects, CPF is also an endocrine disrupting chemical (EDC) affecting the synthesis of some sexual hormones such as testosterone, estradiol and progesterone (Ventura et al. 2016; Chebab et al. 2017). CPF is also able to disrupt the thyroid hormone system (De Angelis et al. 2009), with possible consequences also at neurological level, and to differently affect the gene expression of oestrogen and androgen receptors according to the cellular model used (Grünfeld and Bonefeld-Jorgensen 2004; Venerosi et al. 2015; Coppola et al. 2022; Lori et al. 2024). Administration of CPF to rodents during gestation impaired the reproductive health of both male and female animals (Ubaid Ur Rahman et al. 2021).

In conclusion, the in vivo animal data consistently support the hypothesis that CPF is a developmental toxicant, although such effects have not been completely confirmed in epidemiological studies. The molecular mechanism of developmental toxicity of CPF is unclear, but it is unlikely to be based (or at least exclusively) on AChE inhibition, at least as regards neurotoxicity. Clarifying the molecular mechanisms involved in CPF developmental toxicity is a crucial step in assessing the real risk of developmental toxicity in humans.

Neurodevelopmental and reproductive effects are of primary concern because of their socioeconomic implications. So far, regulatory agencies rely only on animal data to establish reference doses. The Organisation for Economic Co-operation and Development (OECD) has standardised guidelines (Test 426) for testing neurodevelopmental (OECD 2007) as well as reproductive/developmental toxicity (Test 421) (OECD 2016). However, there is no mandatory requirement to apply the test guideline 426, and neurodevelopmental concerns are typically addressed through the results of multigenerational reproductive toxicity studies or other neurotoxicity studies (Terron and Bennekoub 2018). The OECD (https://www.oecd.org/env/ehs/testing/developmental-neurotoxicity.htm) (OECD 2023), EFSA (EFSA Panel on Plant Protection Products and their Residues 2021), European Chemical Agency (ECHA) and other regulatory agencies have proposed strategies to address this gap through careful evaluation of evidence, based on batteries of New Approach Methods (NAMs) and Integrated Approaches to Testing and Assessment (IATAs), considered as priority areas for the Next Generation Risk Assessment (NGRA).

NAMs can play a key role in the study of the molecular mechanisms of toxicity and can significantly contribute to the identification of molecular key events (KEs) leading to adverse outcomes, thus supporting the evidence of Adverse Outcome Pathways (AOPs). Therefore, the use of NAMs contributes to a more efficient prediction of the mode of action of chemicals and pushes forward the advance towards a well-organised and predictive testing strategy.

In this work, we have extensively reviewed the scientific literature to select articles investigating the developmental toxicity of CPF using NAMs. Through an integrative approach, we have collected and evaluated all available information to support regulatory decisions related to CPF using the OECD and EFSA approaches.

## Materials and methods

### Literature sources and search string

Web of Science and PubMed were searched for the literature on 02 May 2023 using the following terms:

“(chlorpyrifos AND development*) AND (in vitro OR alternative OR new approach methodology OR NAM OR cellular system)”

### Inclusion/exclusion criteria

The inclusion/exclusion criteria are summarised in Table 1.

**Table 1:** Inclusion/exclusion criteria for study selection

Inclusion	Exclusion
Studies published since 2000 Studies published in English Research papers Full text papers Based on CPF^a^ Non-epidemiological studies Toxicological endpoints In vitro endpoints^b^ Developmental endpoints	Studies published before 2000 Studies published in languages other than English Reviews Abstracts, communications, etc Based on pesticides other than CPF^a^ Epidemiological studies Non-toxicological endpoints (e.g. efficacy and entomology) In vivo studies^b^ Non developmental endpoints

^a^Studies where CPF was investigated together with other pesticides were included, but only the information on CPF was considered in the assessment

^b^Studies where both in vivo and in vitro approaches were included, but only the information on in vitro results was considered in the assessment

### Literature screening and data extraction

After duplicates removal, the 386 publications potentially suitable for inclusion in this review were analysed using a two-step strategy. At first, title and abstract of each publication were screened according to inclusion/exclusion criteria (Table 1), reducing the number of studies to 94. The full text of these studies was screened again using the same inclusion/exclusion criteria; this excluded 24 additional studies, leaving a total of 70 eligible studies which were grouped according to the biological model species: human, rodent, fish and avian (Supplementary Information 1). One study included both human and rodent models. The following data were extracted from each study and entered into Excel spreadsheets: biological model, CPF exposure (dose/time), endpoint, main conclusion and DOI.

### Quality assessment

In the final stage, we performed an individual quality assessment of the 70 studies identified using the ToxRTool (Toxicological data Reliability Assessment Tool) developed by the EU Joint Research Center (Schneider et al. 2009), in particular, the sheet specific for the assessment of the reliability of in vitro toxicity studies according to Klimisch’s categorisation system (Klimisch et al. 1997). This tool uses a checklist that addresses key aspects such as: (1) identification of the test substance; (2) characterisation of the test system; (3) details of the study design; (4) documentation of the study results; and (5) plausibility of both the design and the results. The checklist requires questions to be answered with either YES (1 point) or NO (0 points). A minimum of 15 points responses is required to receive a Klimisch (K) 1 label (reliable without limitations). Studies with between 11 and 14 points are given a K2 label (reliable with limitations), while those with less than 11 points are given a K3 label (unreliable). Studies with inadequate documentation (e.g. reviews, handbooks or other secondary sources) were rated K4 (not assignable). In addition, certain critical aspects of the study are marked as ‘red questions’ and must be answered YES (1 point), otherwise the study is rated K3.

### AOP network

On the basis of the endpoints assessed in the 70 papers, we searched for the availability of correspondent KEs in the AOP-Wiki collaborative portal (https://aopwiki.org/), identifying four AOPs, namely 405, 475, 499 and 500. The AOP-network finder (https://aop-networkfinder.no/; Yarar et al. 2024), was used to query these AOPs to obtain a network of the featured Molecular Initiating Events (MIEs), KEs and Adverse Outcomes (AOs), which was downloaded and modified within Cytoscape 3.10 (Shannon et al. 2003).

## Results and discussion

### Search results and quality assessment

**Fig. 3:**
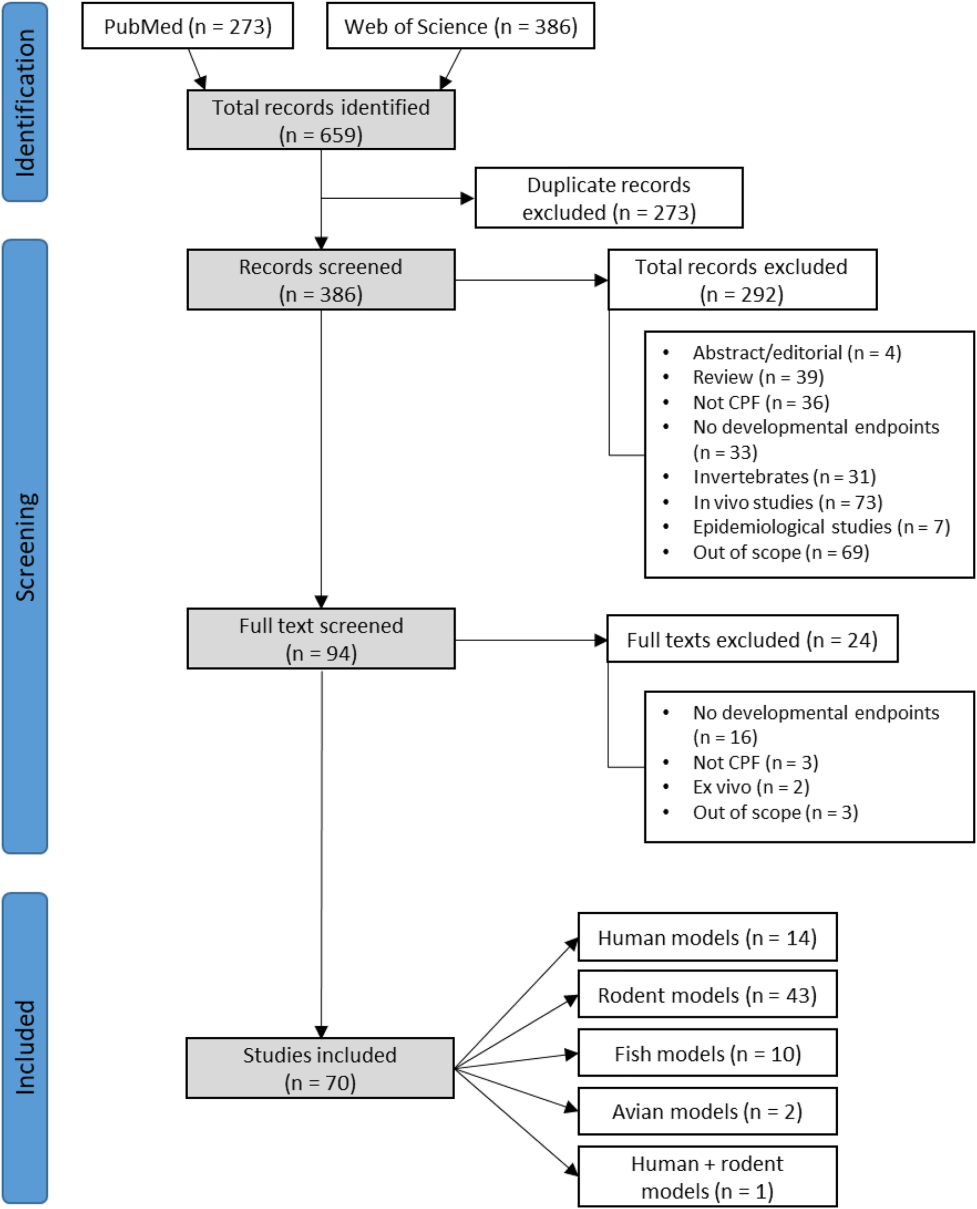
Flow diagram for the identification and selection of the studies included in this review

A strategy and workflow according to the PRIMA statement (Page et al. 2021) was followed, as shown in Fig. 3. A total of 659 studies were retrieved by the search strategy, of which 273 were duplicates. After the initial screening based on title and abstract evaluation, a total of 292 studies were excluded because they met some of the exclusion criteria. Figure 3 shows the number of studies excluded for each reason. Ninety-four records remained and after full text analysis the final number of studies included in the assessment was reduced to 70, which were grouped into four different categories according to the biological model: human cells (14 studies), rodent models (43 studies), fish models (10 studies), avian models (2 studies) and human + rodent models (1 study) (Fig. 3).

Our list of inclusion/exclusion criteria (Table 1) prevented the inclusion of studies with inadequate documentation. Therefore, none of the 70 studies finally included in the assessment had a label K4. All 70 studies received a score higher than 11; specifically, 60 were labelled K1 (reliable without limitations) and 10 were labelled K2 (reliable with limitations). The Klimisch scores and labels for each study are shown in Supplementary Information 1.

### Human models

A total of 15 papers were identified using human models to study the developmental effects of CPF (Supplementary Information 1). In particular, the included studies implied only in vitro models of neurotoxicity, such as neuronal progenitor cells (NPCs) differentiated from induced pluripotent stem cells (iPSCs) or other similar models. Table 2 summarises these studies which are described below by endpoint, including cytotoxicity and apoptosis, inhibition of AChE and glutamate uptake, cytoskeletal changes, oxidative stress, changes in the expression of genes and proteins involved in differentiation, and others.

**Table 2:** Comprehensive summary of adverse effects caused by CPF reported using human cell models

Biological	CPF (exposure)				
model	Dose	Time	Endpoint	Main conclusions	Doi link
Foetal astrocytes (16–19 weeks of gestation)	25–50–100 µM	7 or 14 days	Cell growth, lactate dehydrogenase, glutamate uptake, TUNEL, transcriptomics, gene and protein expression	CPF inhibited astrocyte growth and survival. CPF affected genes critical for neural development and CNS function, the insulin and the IFN-γ-signalling pathways, and inflammation of astrocytes	Mense et al. (2006) http://dx.doi.org/10.1093/toxsci/kfl046
HUCB-NSC	0.01–60 µM	48 h	Cell viability	60 µM CPF reduced to 16% viability	Buzanska et al. (2009) http://dx.doi.org/10.1002/stem.179
Native and differentiated NT2 cells	10 nM–100 µM	24–48–72 h	Cell growth; AChE activity	CPF induced cell death and a dose-dependent inhibition of AChE activity	Amaroli et al. (2013) http://dx.doi.org/10.1016/j.chemosphere.2012.11.005
NPC	7, 14, 29, 43, 57, 143, 285 and 570 µM	24 or 72 h	Cell viability, apoptosis, protein expression and histone modifications	CPF decreased cell number with dose-dependent increase of apoptotic cells. Dose-dependent decrease of β-tubulin III, PCNA and SOX-2 expression. Concentration-dependent decrease of ERK1/2 only under proliferating conditions. Concentration-dependent increase in DMH3Lys4 and reduction in HDAC4	Kim et a. (2016) http://dx.doi.org/10.1016/j.reprotox.2016.08.005
NPC, NeuroNet pure human neurons and AstroPro human astrocyte cells	0.3, 1, 3, 10, and 30 µM	48 h	Immunocytochemistry, metabolomics	CPF inhibited neurites per neuron, neurite length per neuron, and branch points per neuron. Co-culturing with astrocytes rescued neurite number, neurite outgrowth and branch point phenotypes	Wu et al. (2017) http://dx.doi.org/10.1093/toxsci/kfx056
iPSCs and NSC	10 µM	2, 4, 6 days	Gene expression, AChE and butyrylcholinesterase activity	CPF inhibited both esterases but did not affect their mRNA expression as well as HES5, MAP2, and DCX gene expression	Tiethof et al. (2018) http://dx.doi.org/10.3390/toxics6030052
NPC (ReNCell VM)	0.1–50 µM	24 h	Cell viability, DNA damage, mitochondrial membrane potential, apoptosis, and intracellular glutathione levels	CPF decreased cell viability, mitochondrial membrane potential, and intracellular glutathione levels, increasing nucleic DNA damage. An increase in the number of apoptotic cells was noted up to 0.78 µM CPF, after which necrosis is dominant	Mahajan et al. (2019) http://dx.doi.org/10.1007/s00204-019-02549-9
NCS (hNS1)	0.1, 1, 10, 20, 30, 60, 120 and 240 µM	4- and 7-day differentiation	Cell death, proliferation, cell fate specification	CPF increased Caspase 3 activity and number of pyknotic nuclei at 120 and 240 µM. Ki67 and SOX2 were not affected. CPF induced a dose-dependent increase of GFAP (astrocyte marker). GFAP/Ki67 positive cells increased in a dose-dependent manner	Sandoval et al. (2019) http://dx.doi.org/10.1016/j.scitotenv.2019.05.270
NSCs	21, 37.1 µM	14 days	Gene expression of CYP enzymes, alterations in synapses, neurite outgrowth, brain-derived neurotrophic factor, neurons/astrocytes, and electrical activity	Upregulation of some cytochrome P450 and glutathione S-transferase genes during neuronal differentiation; formation of the two major CPF metabolites (due to bioactivation and detoxification) supporting the metabolic competence of the model. Alterations in the number of synapses, neurite outgrowth, BDNF, proportion of neurons and astrocytes, as well as of electrical activity	Di Consiglio et al. (2020) http://dx.doi.org/10.1016/j.reprotox.2020.09.010
iPSC (253G1) differentiated into NPC	1–10–100–1000 µM	48 h	Cytotoxicity (MTS and ATP assays)	CPF induced cytotoxicity in a dose-dependent manner at lower concentrations in iPSC than in NPC	Kamata et al. (2020) http://dx.doi.org/10.1016/j.tiv.2020.104999
NSCs originally derived from iPSC (IMR90)	0.49, 1.95, 7.81, 31.25, 125 and 500 µM	3 or 14 days	Cell viability, synaptogenesis, neurite outgrowth, BDNF protein level	SYP was increased after 3-day treatment, while at 14 days decreased at pre-synaptic neurite level and post-synaptic PSD95 was increased. Total levels of BDNF increased after 3 and 14 days	Pistollato et al. (2020) http://dx.doi.org/10.1186/s12940-020-00578-x
iPSC-derived 3D brain neurospheres (CHD8^+/+^ and CHD8^±^)	47 and 100 µM	24 h at both 4 and 8 weeks of differentiation	Cytotoxicity, mitochondrial membrane potential, ROS production, immunofluorescence, qPCR, AChE activity, neurite outgrowth, WB	Higher levels of ROS in CHD8^±^ than in CHD8^+/+^ spheroids after CPF exposure. CPF decreased AChE activity at similar levels in both cell lines. BrainSpheres exposed to CPF had significantly shorter neurite length	Modafferi et al. (2021) http://dx.doi.org/10.1289/EHP8580
NPCs	0.3 and 15 µM	5 and 10 days	Cell viability, RNA-seq	No cytotoxicity. CPF affected neurite outgrowth GO terms	De Leeuw et al. (2022) http://dx.doi.org/10.1016/j.chemosphere.2022.135298
iPSCs (IMR90) differentiated into 2D and 3D NSC mixed cultures of neurons and glial cells	0.12, 0.49, 1.95, 7.81, 31.25, 125, and 500 µM	7 days	Cell viability and qPCR	In 2D, *GRIA1* gene expression was upregulated by CPF. In 3D, *GABRB3* was decreased, while it was upregulated in 2D. *SLC18A3* was significantly repressed by CPF in 3D. *NEUROD1* was increased in 2D and 3D cultures. *ERBB4* was decreased only in 3D. *NTRK1* was downregulated by in 3D and increased in 2D cultures	Nunes et al. (2022) http://dx.doi.org/10.1016/j.reprotox.2022.03.017
LUHMES cells	100 nM–300 µM	24 h	Caspase 3 activity, DNA fragmentation, neurite length,	Apoptosis, neurite shortening, ROS increase, loss of MMP and caspase 3 activation	Singh et al. (2018)^a^ http://dx.doi.org/10.1016/j.nbd.2018.05.019

^a^This study is mixed human + rodent; therefore, it is also mentioned in Table 3

#### Cytotoxicity and apoptosis

The cytotoxicity of CPF was assessed in most studies, either as a screening for a battery of chemicals or as a prelude to subsequent assessments, using a variety of assays. A comparison of CPF cytotoxicity on iPSC and NPC was performed in the concentration range of 1–1000 µM, showing lower IC_50_ (concentration that reduces cell viability by 50%) values in iPSC after 48-h treatment (IC_50_ = 86.1 ± 34.9 µM for MTS assay and 19.9 ± 2.3 µM for ATP assay) compared to NPC (IC_50_ = 217 ± 57 µM for MTS assay and 136 ± 38 µM for ATP assay) (Kamata et al. 2020). The viability of human NPC was differentially reduced by CPF depending on the growth condition; in the range of 7–570 µM, CPF reduced the viability of proliferating cells after 24-h treatment (IC_50_ = 171 µM), whereas at 72 h both proliferating and differentiating cells were affected, the latter being more sensitive (IC_50_ = 152 µM and 71 µM, respectively). However, at 57 µM and above, proliferating cells had a higher proportion of dead and non-apoptotic cells compared to differentiating cells (Kim et al. 2016).

In another study, cell viability of iPSC-derived neuronal stem cells (NSC) grown in differentiating conditions (21 days) and treated with CPF in a repeated dose regimen (twice/week) was reduced only at the two highest concentrations tested, 125 and 500 µM (Pistollato et al. 2020). Conversely, after 2 weeks of differentiation under the same treatment conditions, CPF did not significantly affect NSC viability up to 500 µM, where a decrease of about 40% was observed in both 2D and 3D culture conditions (Nunes et al. 2022). 3D culture was also applied to NPC containing neurons, astrocytes and oligodendrocytes, called BrainSpheres; no effect on cell viability was observed after 24-h treatment with CPF at 47 and 100 µM, after 4 or 8 weeks of differentiation (Modafferi et al. 2021). Similarly, CPF did not affect the expression of Ki67, a marker of proliferation, up to 240 µM in hNS1 cells, a multipotent stem cell line derived from the telencephalic region of the developing human brain, under differentiating conditions (5 days). However, a dose-dependent increase in caspase 3 protein expression, significant at 120 and 240 µM, and an increased number of pycnotic nuclei, which occur when chromatin condenses during apoptosis, were observed at the same concentrations in the same cells (Sandoval et al. 2019). Apoptosis was also reported in Lund human mesencephalic (LUHMES) neuronal cells, where CPF caused a dose-dependent increase (IC_50_ = 1.3 µM) of caspase 3 activation (Singh et al. 2018).

In ReNcell VM cells, an immortalised human NPC line, CPF appeared more toxic compared to the other cells described above, as it decreased cell viability after 24-h treatment (range 0.1–50 µM) in a dose-dependent manner, with an IC_50_ ≈ 9.9 ± 0.17 µM. Furthermore, the number of apoptotic cells increased up to 0.78 µM, after which necrosis predominated (Mahajan et al. 2019). Conversely, CPF reduced the viability of human umbilical cord blood-derived NSC (HUCB-NSCs) at an early developmental stage by 84%, but only at 60 µM. At the same concentration, the viability of spontaneously differentiating HUCB-NSCs (2 weeks) and cells induced to differentiate into neurons or astrocytes was reduced by 19 and 55%, respectively (Buzanska et al. 2009). Similarly, NT2 neuronal cells differentiated from the NTERA-2 cell line, derived from a human male germ cell carcinoma, showed a reduction in growth of 18, 40 and 63% at 24, 48 and 72 h, respectively, after treatment with CPF at 100 µM (Amaroli et al. 2013). At lower concentrations (30 µM), CPF reduced the cell density of hNP neurons after 28 days in vitro (DIV), as evidenced by MAP2 (microtubule-associated protein 2) positivity (Wu et al. 2017).

CPF at 25 and 50 µM decreased the cell number of human primary foetal astrocytes (from elective abortions in the second trimester) after 14 (but not 7) days of exposure. Consistently, CPF dose-dependently increased LDH activity in the range of 0.2 to 25 µM after 14 days of exposure; however, apoptosis was only observed at 100 µM (Mense et al. 2006).

Overall, these results showed that ReNcell VM were the most sensitive cells to CPF exposure and iPSCs to be more sensitive than NPCs. Furthermore, the viability of NPCs was influenced by the growth conditions and duration of exposure, with differentiating cells showing greater sensitivity over time. This suggests that the developmental stage of the cells may influence their susceptibility to chemical insults. The considered studies varied in their evaluation methodologies; however, most of them detected significant cytotoxicity of CPF in the micromolar range.

#### Inhibition of AChE activity and glutamate uptake

The main mode of action of CPF is as inhibitor of AChE, but the activity of this enzyme was only measured in three studies. CPF induced a dose-dependent inhibition of AChE activity in the range of 1–100 µM in NT2 cells. While differentiated cells treated with CPF showed AChE immunolocalisation on the membrane, as in control cells, undifferentiated treated cells showed more relevant perinuclear, cytoplasmic and membrane staining compared to controls (Amaroli et al. 2013). In NPC BrainSpheres at 4 weeks of differentiation, CPF at 100 µM decreased AChE activity by 41%, but not gene expression. When cells were heterozygous knocked out for *CHD8*^+^ (CHD8^±^) (a gene highly correlated to the risk of autism), AChE activity was reduced to 37%. A moderate and nonsignificant increase in acetylcholine (ACh), but not choline, levels was also observed in both cell genotypes (Modafferi et al. 2021). Both AChE and butyrylcholinesterase activities were decreased by 82% and 92%, respectively, by CPF at 10 µM in NSC during differentiation up to 6 days of exposure. No parallel inhibition of gene expression was observed (Tiethof et al. 2018).

One study investigated the effect of CPF exposure on glutamate uptake, a relevant mechanism regulating neurotransmission, in human primary foetal astrocytes, which was decreased in a dose-dependent manner in the range of 1 to 25 µM (Mense et al. 2006).

These results suggest that even low concentrations of CPF can disrupt cholinergic signalling, which is crucial for neuronal communication. In addition, the significant reduction of glutamate uptake in primary human foetal astrocytes further indicates the broader impact CPF may have on neurotransmitter regulation.

#### Neurite outgrowth

Neurite morphology and cytoskeletal organisation were assessed in six studies. A decrease in neurite outgrowth, including neurite length, branch points and neurite/neuron ratio, was observed in NSC after 3 and 14 days of treatment with CPF at 37.1 µM under differentiating conditions. In addition, β-III-tubulin immunoreactivity (IR) increased at the same concentration (Pistollato et al. 2020). This result was confirmed by a subsequent study showing a dose-dependent decrease in neurite outgrowth, in particular the neurite/neuron ratio and the number of branching points, in the same cells after 14 days of treatment with CPF (18.45–37.1 µM) under differentiating conditions. However, neurite length was not significantly affected (Di Consiglio et al. 2020). Contrarily, Singh and co-workers (2018) reported neurite shortening in LUHMES cells after exposure to 300 nM CPF during 24 h. At 10 µM, CPF inhibited neurite/neuron ratio, neurite length and branching in neurons (DIV 28); the effects were rescued when neurons were co-cultured with astrocytes, possibly through their P450 activity (Wu et al. 2017). Under differentiation conditions, exposure to CPF at 57 µM inhibited neurite outgrowth and clustering of human NPC (Kim et al. 2016). At a higher concentration (100 µM), CPF also significantly inhibited neurite outgrowth in NPC BrainSpheres after 4 weeks of differentiation (Modafferi et al. 2021). An additional effect was observed in ReNcell VM cells with changes in cytoskeletal organisation, actin downregulation and cell area shrinkage with increasing CPF concentration (range 0.1–50 µM) and exposure time (Mahajan et al. 2019).

In general, these studies highlight the neurotoxic effects of CPF on neurite outgrowth, demonstrating consistent reductions in key metrics such as neurite length and branching in various cell types.

#### Oxidative stress

Effects on intracellular reactive oxygen species (ROS) and glutathione levels, as well as on mitochondrial membrane potential (MMP), as markers of oxidative stress induction, were assessed in three studies. ROS levels and MMP were measured in BrainSpheres of NPCs treated with CPF 100 µM for 24 h after 4 weeks of differentiation and no change was observed (Modafferi et al. 2021). Conversely, CPF induced a dose-dependent decrease in MMP, intracellular glutathione levels and DNA staining in the nuclei of ReNcell VM cells, indicating damage (IC_50_ ≈ 5.5 ± 0.23 µM; IC_50_ ≈ 8.25 ± 0.25 µM; IC_50_ ≈ 11.6 ± 0.45 µM, respectively) (Mahajan et al. 2019). The loss of MMP and ROS activation was also reported in LUHMES cells after 24 h of exposure at 300 nM CPF (Singh et al. 2018).

Overall, CPF demonstrated to induce oxidative stress in at least two developmental models, with BrainSpheres appearing insensitive to this effect.

#### Gene and protein expression

Nine studies investigated changes in gene and/or protein expression induced by CPF exposure. In particular, CPF at 10 µM did not affect the gene expression of *MAP2*, *DCX* and *HES5* in NSC up to 6 days of differentiation (Tiethof et al. 2018). Similarly, no effect on gene expression of the stem cell marker SOX2 was observed up to 240 µM in hNS1 cells after 5 days of differentiation (Sandoval et al. 2019). *SOX2* expression was also unaffected by CPF (2.5–9.8 µM) at the protein level, as observed by immunofluorescence in ReNcell VM (Mahajan et al. 2019). However, in another study, exposure of human NPC to CPF for 72 h revealed a concentration-dependent decrease in the protein expression of SOX2, as well as the neuronal marker β-III-tubulin and the proliferating cell nuclear antigen (PCNA), significant at 57 µM in both proliferating and differentiating conditions. A dose-dependent decrease in p42/44 MAPK (ERK1/2) protein expression was also observed only in proliferating conditions and was significant at 57 µM (Kim et al. 2016). Contrary to previous findings, the protein expression of β-III-tubulin was not affected by CPF in hNS1 cells under differentiating conditions (5 days); however, in the same cells, CPF induced the gene and protein expression of GFAP, an astrocyte marker, in a dose-dependent manner, which was significant from 60 to 240 µM, supporting the promotion of gliogenesis, confirmed also by the increase in the number of double-positive GFAP^+^ and Ki67^+^ cells (Sandoval et al. 2019). A significant increase in GFAP protein expression was also observed in human primary foetal astrocytes exposed to CPF at 5 and 25 µM for 1 week; CPF also significantly increased IL-6 protein expression and activated (phosphorylated) ERK1/2 (Mense et al. 2006).

An increase in SYP (presynaptic marker) was observed in NSC after 3 days of treatment with CPF at 37.1 µM under differentiating conditions; after 14 days, a decrease in SYP IR was observed at the neurite level, with a parallel increase in PSD95 IR (post-synaptic marker) at 21 and 37.1 µM, with a resulting decrease in SYP/PSD95 (synapses) co-localisation evident at 37.1 µM. Brain-derived neurotrophic factor (BDNF) IR was increased by CPF at 3 and 14 days at 37.1 µM, with a parallel decrease in neurite to cell body ratio for BDNF levels (Pistollato et al. 2020). A similar dose-dependent decrease in the co-localisation of SYP/PSD95-positive cells was induced by CPF (18.45–37.10 µM) in NSC after 14 days of treatment under differentiating conditions. The increase in BDNF IR was also confirmed by a dose-dependent trend in the same dose range and conditions. In addition, CPF induced a modest increase in the percentage of neuronal cells (10–15%), as evidenced by β-III-tubulin-positive cells, but a consistent dose-dependent increase in GFAP-positive cells, indicative of astrocytes (Di Consiglio et al. 2020).

At 100 µM, CPF did not affect gene expression of CHD8 in NPC BrainSpheres at 4 weeks of differentiation; however, CHD8 protein expression was reduced by 80%. In addition, gene expression of tyrosine hydroxylase (TH), an enzyme responsible for the conversion of tyrosine to L-DOPA, was higher in CPF-treated BrainSpheres (Modafferi et al. 2021). Several genes were investigated in NPC differentiated for 2 weeks and treated for 7 days; in both 2D and 3D culture conditions, CPF (0.12–125 µM) did not affect the gene expression of *GRIA2*, *GRIA3* and *GAP43* (glutamatergic neuron-related genes), *GABRA3* (GABAergic neuron), *TH* and *NR4A2* (dopaminergic neuron-related genes) and *CHAT* and *SLC5A7* (cholinergic genes). *GRIA1* was induced by CPF only in 2D conditions at 125 µM, whereas *GABRB3* was induced in 2D at both 31.25 and 125 µM and repressed in 3D at the lowest concentration tested (0.12 µM). Interestingly, both *SLC18A3*, which encodes the vesicular ACh transporter protein, and *OLIG1*, the oligodendrocyte transcription factor 1, were repressed in 3D by CPF at all concentrations tested. Among the oligodendrocyte markers tested, *PPARγ* was significantly induced only in 2D conditions at 1.95 µM; in addition, in the same culture conditions, *GFRA1* and *BMPR2* were repressed and induced, respectively, at the highest concentration tested (125 µM). Among the NSC genes examined (*NES*, *NEUROD1*, *PAX6* and *RHOA*), only *NEUROD1* was induced by CPF at the lowest concentration (0.12 µM), in both 2D and 3D conditions. Genes related to synaptogenesis were not affected by CPF exposure, except for *ERBB4*, which was decreased at 7.81 µM in 3D. Finally, among the *BDNF-CREB* genes analysed, only *NTRK1* was downregulated in 3D at 1.95 and 7.81 µM, whereas it was upregulated in 2D at 1.95 µM (Nunes et al. 2022).

Interestingly, the expression of some genes encoding P450 enzymes was assessed in NSC to identify changes in cell metabolism; CPF at 21 µM induced *CYP3A5* and *CYP1A1* expression after 24 h and 6 h, respectively. In addition, *GSTA4* was reduced after 6 h. At the same concentration, but under repeated treatment conditions, CPF induced only *CYP2C19* at 1 h, but significantly decreased *GSTM1* at 3, 6 and 24 h, *GSTM3* at 1, 6 and 24 h and *GSTA4* at 3 and 6 h (Di Consiglio et al. 2020).

Another study investigated whether CPF could induce some histone modifications in human NPC. CPF induced a concentration-dependent increase in the protein expression of phosphorylated serine 10 in histone H3 (pAH3Ser10), a marker of apoptosis, significant at 57 µM, in proliferating but not in differentiating cells. One of the enzymes responsible for histone modifications, HDAC4, was decreased in a dose-dependent manner being significant at 57 µM only under differentiating conditions. CPF also induced a significant concentration-dependent increase in di-methylated lysine 4 of histone H3 (DMH3Lys4), significant at 57 µM, in both proliferating and differentiating conditions, which may affect the transcription of genes involved in neurodevelopment and differentiation (Kim et al. 2016).

Two transcriptomic studies were also performed using different approaches. The effects on human primary foetal astrocytes (from elective second trimester abortions) exposed to CPF at 25 µM for 1 week were investigated by microarray analysis, revealing a general inductive effect on gene expression. Among others, CPF affected genes related to central nervous system (CNS) development, insulin signalling and the IFN-γ pathway (Mense et al. 2006). An RNA-Seq approach was used to investigate the transcriptome of human neuron-astrocytes differentiated from NPC and treated with CPF at 0.3 and 15 µM. At both doses, CPF enriched 40 and 113 gene ontology terms, respectively, including differentiation, metabolism, transport, behaviour, apoptosis, migration, proliferation and stress. Among the compounds assessed in the study, a specific effect of CPF on a gene ontology term related to neurite outgrowth was observed (de Leeuw et al. 2022).

Concluding, some of the reported studies highlight early changes in expression determined by CPF exposure supporting the promotion of the astrocyte phenotype, imbalance of the pre–post-synaptic markers and of some neurotransmitters in neurons, and impairment of neurite outgrowth.

#### Other endpoints

One study investigated the changes in the levels of some metabolites in NPC BrainSpheres after 4 weeks of differentiation, following 24-h treatment with CPF 100 µM. Folic acid was found to be increased in both wild-type and CHD8^±^ cells, whereas lactic acid and tryptophan were increased only in CHD8^±^ cells. Glutamate and GABA levels were not affected, whereas the glycine/GABA ratio decreased in CHD8^±^ cells upon CPF exposure. Arginine and ornithine levels were higher in CHD8^+/+^ cells (Modafferi et al. 2021).

Some biomechanical properties were investigated in ReNcell VM after exposure to CPF (2.5, 4.9 and 9.8 µM) at different time points (4, 12, 24 and 36 h). A concentration- and time-dependent decrease in elastic modulus, adhesion force, tethering force, and apparent membrane tension was observed, with a concomitant increase in tethering radius (Mahajan et al. 2019).

Electrical activity was also examined in a study in which NSCs were treated with CPF at 21 µM for 7 days under differentiating conditions. A modest decrease in spike rate and number of bursts was observed; however, a significant decrease in the number of synchronised network bursts was recorded after 10 and 14 days of treatment, indicating an impairment in the formation of the mature neuronal network (Di Consiglio et al. 2020).

The investigation of these endpoints further substantiates the effects of CPF on structural and functional integrity of neurons, with alteration of electrical and mechanical properties, as well as metabolome, also in the low micromolar range.

#### Overall conclusions about human models

In conclusion, the effects of CPF on neurodevelopment have been studied in human cellular models using NAMs, focussing on NPCs NSCs and iPSCs. Across various studies, CPF exposure has shown a wide range of effects, including cytotoxicity, apoptosis, inhibition of AChE activity, glutamate uptake disruption, and oxidative stress induction (Table 2). CPF also impacts key proteins and genes involved in neurodevelopment, such as SOX2, β-III-tubulin, while promoting gliogenesis to neurogenesis impairment (Kim et al. 2016; Sandoval et al. 2019; Di Consiglio et al. 2020). Moreover, CPF influences epigenetic regulation, altering the proteins expression involved in histone modification, such as HDAC4 and DMH3Lys4, as well as proteins like ERK1/2, which are critical to cell differentiation processes (Mense et al. 2006; Kim et al. 2016). Experimental evidence indicates that CPF impairs neurogenesis, resulting in altered neurite outgrowth, overall neural structure and a lower neurite/cell ratio (Pistollato et al. 2020; Di Consiglio et al. 2020; Wu et al. 2017; Kim et al. 2016; Modafferi et al. 2021; Mahajan et al. 2019).

These alterations highlight the ability of CPF to interfere with neuronal network development and synaptogenesis, potentially contributing to long-term neurodevelopmental deficits. Overall, the results demonstrate that CPF has a significant impact on neurodevelopment, with human-based NAMs playing a key role in clarifying the mechanisms of action of CPF.

#### Rodent cells

A total of 44 papers were identified that used rodent models to study the developmental effects of CPF (Supplementary Information 1). The models comprised mainly PC12 cells, embryonic stem cells and various primary (cortical, sympathetic, telencephalic neurons, etc.) and cancer cells (glioblastoma, neuroblastoma, etc.). Table 3 summarises the different endpoints and main conclusions of all these 44 studies.

**Table 3:** Comprehensive summary of adverse effects caused by CPF reported using rodent cell models

Biological	CPF (exposure)				
model	Dose	Time	Endpoint	Main conclusions	Doi link
Undifferentiated and under differentiation PC 12 cells	50 µM	2 and 6 days	AC signalling cascade	2-day exposure in differentiating cells inhibited AC measures when exposure commenced at the initiation of differentiation. The effect on AC is noted in a discrete window of time at the beginning of the neurodifferentiation. No effect on undifferentiated cells	Adigun et al. (2010) https://doi.org/10.1016/j.brainres.2010.03.025
Undifferentiated and under differentiation PC 12 cells	30–50 µM	6 days	DNA synthesis, cell number and size, and cell signalling mediated by AC	Cholinergic hyperstimulation, oxidative stress, and interference with AC signalling contributes to the developmental neurotoxicity of CPF	Slotkin et al. (2007a) http://dx.doi.org/10.1289/ehp.10194
Undifferentiated and under differentiation PC 12 cells	Up to 50 µg/mL	10 min–72 h	Oxidative stress	CPF during 10 min increased ROS concentration. CPF did not elicit significant ROS increase in undifferentiated PC12 exposed 72 h. This exposure (72 h) inhibited neurite outgrowth in differentiating cells but not significant variation in ROS production were noted. CPF can elicit acute (but not chronic) increases in ROS production	Crumpton et al. (2000a) http://dx.doi.org/10.1016/S0165-3806(00)00045-6
Undifferentiated and under differentiation PC 12 cells	30 µM	1 h, 1 day or 6 days	DNA synthesis and lipid peroxidation	CPF inhibited DNA synthesis in undifferentiated PC12 cells. CPF increased lipid peroxidation in undifferentiated cells after 24 h of exposure. CPF inhibited DNA synthesis in differentiating cells after 6 days of exposure. This same exposure increased the total protein/DNA ratio and did not alter the membrane/total protein ratio	Lassiter et al. (2009) http://dx.doi.org/10.1016/j.brainresbull.2008.09.020
Undifferentiated and under differentiation PC 12 cells	0.1–100 µM	24 h	Oxidative stress	CPF achieved significant oxidative stress. Similar results were noted for differentiated PC12 cells	Qiao et al. (2005) http://dx.doi.org/10.1016/j.taap.2004.11.003
Undifferentiated and under differentiation PC 12 cells	30 µM	24–72 h	Transcriptional profiles for genes for oxidative stress responses and glutamate receptors	Undifferentiated cells: Significant but modest effects on genes mediating antioxidant responses and glutathione metabolism. Consistent increases for the glutamate receptors *Gria1*, *Grik4*, *Grik5*, *Grin3a*, and *Grina* and persistent decreases for *Gria2*, *Gria4*, and *Grik2* Differentiating cells: Greater overall response for the genes involved in antioxidant activity than in non-differentiating cells. Approximately, the same net absolute response for glutamate receptor genes as in undifferentiated cells but with increases in *Grid2*, *Grin1*, and *Grin2b* and decreases in *Grin3b* restricted to differentiating cells	Slotkin and Seidler (2009b) http://dx.doi.org/10.1289/ehp.0800251
PC 12 cells under differentiation	30–50 µM	6 days	Lipid peroxidation, DNA content, total protein/DNA	CPF evoked oxidative stress (lipid peroxidation). Ascorbate provided protection to reduce MDA, whereas NGF did not. Neither ascorbate nor excess NGF provided protection against the loss of cells caused by CPF. The increase in cell size caused by CPF was not reduced by ascorbate or higher NGF	Slotkin and Seidler (2010b) http://dx.doi.org/10.1016/j.ntt.2009.12.001
Undifferentiated and under differentiation PC 12 cells	30 µM	24 and 72 h	Transcriptional profiles related to differentiation into ACh, dopamine and norepinephrine phenotypes	CPF evoked a pattern of effects on genes involved in neural growth and neurite extension and promoted differentiation into the dopamine phenotype at the expense of the ACh phenotype. The transcriptomic profiles readily explain many of the outcomes from exposure to CPF in vivo	Slotkin and Seidler (2009a) http://dx.doi.org/10.1016/j.brainresbull.2008.08.021
Undifferentiated and under differentiation PC 12 cells	30 and 50 µM	6 days	DNA synthesis, cell number and size, and cell signalling mediated by AC	Cholinergic receptor antagonists had little or no protective effect against reduction in DNA synthesis induced by CPF in undifferentiated cells. However, the antagonists showed partial protection against deficits in cell loss and alteration in cell size elicited by CPF in differentiating cells but were ineffective in preventing the impairment of AC signalling	Slotkin et al. (2007b) http://dx.doi.org/10.1289/ehp.9527
Undifferentiated and under differentiation PC 12 cells	5 or 50 µM	3 or 7 days	DNA synthesis, expression of tyrosine hydroxylase, expression of choline acetyltransferase	Undifferentiated cells: CPF significantly reduced DNA synthesis without eliciting cytotoxicity. CPF increased the expression of TH without affecting choline acetyltransferase Differentiating cells: CPF at the start of differentiation significantly reduced choline acetyltransferase but not TH activities. CPF added 4 days after the neurodifferentiation begun, choline acetyltransferase was unaffected, and TH was increased slightly	Jameson et al. (2006) http://dx.doi.org/10.1289/ehp.8750
PC12 cells under differentiation	30 µM	48 h	Expression of gene encoding for different AChE isoforms	CPF enhanced gene expression for AChE-R and AChE-S	Jameson et al. (2007) http://dx.doi.org/10.1289/ehp.9487
Undifferentiated and under differentiation PC 12 cells	30 µM	24–72 h	Expression of genes encoding glutamate transporters	CPF had a greater effect in cells undergoing neurodifferentiation as compared to undifferentiated PC12 cells. A peak sensitivity (global upregulation of all the glutamate transporter genes) was noted at the initiation of differentiation	Slotkin et al. (2010) http://dx.doi.org/10.1016/j.brainresbull.2010.06.010
PC12 cells under differentiation	10–50 µM	6 days	TH and choline acetyltransferase activities, total amount of DNA, protein, and membrane protein	CPF had a promotional effect on neuritogenesis. The effect of CPF on differentiation into specific neurotransmitter phenotypes was shifted by dexamethasone. In dexamethasone-primed cells, CPF enhanced cholinergic neurodifferentiation instead of suppressing this phenotype	Slotkin et al. (2012) http://dx.doi.org/10.1016/j.ntt.2012.07.002
Undifferentiated and under differentiation PC 12 cells	30 µM	24–72 h	mRNAs encoding for serotonin biosynthesis, storage, and degradation, as well as serotonin receptors	In both undifferentiated and differentiating cells, CPF induced tryptophan hydroxylase, the rate-limiting enzyme for serotonin biosynthesis and suppressed expression of serotonin transporter genes, effects that would tend to augment extracellular 5HT Serotonin receptors: in both cellular states CPF increased the expression of *Htr1a*, *Htr1d*, *Htr2b* and *Htr5a* genes and decreased the expression of *Gtr3b*. *Htr1b* and *Htr6* were overexpressed only in differentiating cells, the expression of htr5b was stimulated in undifferentiated cells only; while a small but significant decrease in the expression of htr7 gene was restricted to differentiating cells	Slotkin and Seidler (2008) http://dx.doi.org/10.1016/j.taap.2008.08.020
PC12 cells under differentiation	30 µM	24–72 h	Variations in mRNA of 40 genes encoding for neurotransmitters and circuits	CPF evoked robust upregulation of cholecystokinin, corticotropin-releasing hormone, galanin, neuropeptide Y, neurotensin, pre-proenkephalin and tachykinin 1. Neuropeptides are likely to be a prominent target for the developmental neurotoxicity of CPF	Slotkin and Seidler (2010a) http://dx.doi.org/10.1016/j.brainres.2010.07.073
PC12 cells under differentiation	9, 29, 86, 171 µM	5 days	Outgrowth of neurites and transcriptional alterations of selected genes	CPF induced dose-dependent neurite outgrowth. Correlation with upregulation of *Gap-43*	Christen et al. (2017) http://dx.doi.org/10.1016/j.taap.2017.03.027
Undifferentiated and under differentiation PC 12 cells	50 µg/mL	48 h	Effect on nuclear transcription factors involved in cell replication and differentiation	CPF reduced the expression of the transcription factor Sp1 and AP-1 in PC12 cells under neurodifferentiation. In undifferentiated PC12 cells, CPF reduced the expression of the transcription factor Sp-1, but not the expression of AP-1	Crumpton et al. (2000b) http://dx.doi.org/10.1016/S0006-8993(99)02357-4
Undifferentiated and under differentiation PC 12 cells	30 µM	24–72 h	mRNA levels of genes encoding for subtypes of protein kinase C and their modulators	Undifferentiated cells: significant upregulation of 3 subtypes of protein kinase C as well as one of the regulatory binding proteins Differentiating cells: significant net upregulation of protein kinase C genes	Slotkin and Seidler (2009c) http://dx.doi.org/10.1016/j.brainres.2009.01.049
PC12 cells under differentiation	30 µM	24–72 h	Alterations in transcription of Parkinson’s disease-related genes	Time-dependent changes in gene expression that were not dependent on the differentiation state of the cells. Initially, there were no significant overall changes but by 72 h there were gene-specific effects. Two genes were robustly upregulated (*Acmsd* and *Snca*), whereas four others showed significant decrements (*Atp13a*, *Bst1*, *Gba3*, *Lamp3*)	Slotkin and Seidler (2011) http://dx.doi.org/10.1016/j.brainresbull.2011.09.017
Undifferentiated and under differentiation PC 12 cells	30 µM	24–72 h	Alterations in mRNAs levels of genes encoding for apoptosis a cell cycle	The genes affected in undifferentiated cells were not concordant with those in differentiating cells. The PC12 cell model identified 60–70% of the genes affected by CPF in vivo, indicating that the effects are exerted directly on developing neural cells. These effects do not reflect actions on AChE and operate at exposures below the threshold for any detectable inhibition of this enzyme	Slotkin and Seidler (2012) http://dx.doi.org/10.1016/j.ntt.2011.12.001
Undifferentiated and under differentiation PC 12 cells	30 µM	24–72 h	Expression of 32 mRNAs encoding the neurotrophins, brain-derived neurotrophic factor, NGF, the *Wnt* and *Fzd* gene families and corresponding receptors	Effects in PC12 cells mirrored in vivo effects as regard *Fgf*, *Ntf*, *Wnt* and *Fzd* family expression, especially during early differentiation. Actions on neurotrophic factors provide a mechanism for the developmental neurotoxicity of low doses of organophosphates	Slotkin et al. (2008) http://dx.doi.org/10.1016/j.brainresbull.2008.01.001
D3 mouse embryonic stem cells	0–10000 µM	From differentiation day 1 to day 14	Differentiation into neural cell types	CPF inhibited the formation of MAP2-positive cells. The effects on viability in differentiating cells occurred at similar concentrations, while undifferentiated cells were affected at higher concentrations	Visan et al. (2012) http://dx.doi.org/10.1016/j.neuro.2012.06.006
Rat neural stem cells from embryonic day 14	0–30 µM	3 days/6 days	Differentiation into neuronal and glial phenotypes	CPF suppressed expression of the glial phenotype while the neuronal phenotype was enhanced up to 20 µM	Slotkin et al. (2016) http://dx.doi.org/10.1016/j.tox.2016.10.015
D3 mouse embryonic stem cells	0–1200 µM	12 h/3 days	Gene expression of mouse stem cells	The expressions of *Flk1*, *Oct4* and *AChE* gene were statistically reduced by 40%, 80% and 80%, respectively, while the expression of *Pnpla6* was increased by 70%	Estevan et al. (2013) http://dx.doi.org/10.1016/j.toxlet.2012.11.026
Neurons of mouse adipose tissue-derived stem cells (ADSCs)	0–500 µM	48 h	Expression of neuron-specific genes and proteins	CPF upregulated the expression of some neuron-specific genes (e.g. *Nestin*, *NeuN*, *NEFL*, and *Syp*) during differentiation and seemed to decrease the number of β-tubulin III and MAP2 proteins expressing cells, through mechanisms independent from acetylcholinesterase activity	Zarei et al. (2015) http://dx.doi.org/10.1002/tox.22155
N27 rat embryonic mesencephalic dopaminergic neural cells	100 nM–300 µM	24 h	Immunocytochemistry; caspase 3 activity; GSH and MTS assays; mitochondrial membrane potential, ATP production, oxygen consumption; autophagy	Dose-dependent decrease of N27 cell viability in cell viability and increase in DNA fragmentation. Time-dependent caspase 3 activation, PARP cleavage, ROS and mitochondrial superoxide levels. Bax and Bcl-2 inhibition. Reduction in basal respiration and ATP production. Increase in the release of cytochrome c from mitochondria into the cytosol and in the expression of markers of autophagy	Singh et al. (2018)^a^ http://dx.doi.org/10.1016/j.nbd.2018.05.019
Cortical neurons from foetal Wistar rats (foetal day 18)	100 pM–100 µM	At least 20 min	Spontaneous electrical activity	CPF significantly inhibited the number of spikes, number of bursts/min, mean burst duration and percentage of spikes in burst at concentrations between 21 µM and 34.9 µM	Alloisio et al. (2015) http://dx.doi.org/10.1016/j.neuro.2015.03.013
Rat cortical neurons from embryonic day 17 or new-born rats	0–80 µM	24 h, 48 h, 72 h	Apoptosis	CPF disrupts mitochondrial function and induces apoptosis. Embryonic neurons were more sensitive than postnatal neurons to CPF	Caughlan et al. (2004) http://dx.doi.org/10.1093/toxsci/kfh038
Cortical cells isolated from neonatal rats (postnatal day 0–1)	0.1–10 µM	30 min/14 days	Spontaneous electrical activity of primary cortical cultures	Inhibition of the development of spontaneous electrical activity was observed when cultures were exposed for 14 days to CPF	Dingemans et al. (2016) http://dx.doi.org/10.1016/j.neuro.2016.10.002
Cortical cells isolated from neonatal rats (postnatal day 0–1)	1–100 µM	48 h/14 days	Microelectrode array measurements	Acute exposure resulted in a concentration-dependent decrease in number of spikes (IC_50_ = 16.7 µM), bursts (IC_50_ = 40.4 µM) and network bursts (IC_50_ = 12.5 µM). Inhibition of AChE did not play a role in the observed decrease in neuronal network activity. Exposure (10 days) to 10–100 µM resulted in a significant concentration-dependent decrease in network activity on all three parameters. Exposure (10 days) to 0.1 µM significantly decreased only the number of network bursts	Van Melis et al. (2023) http://dx.doi.org/10.1016/j.neuro.2022.11.002
Mouse cultured cerebellar granule neurons from PND 6–7	1–1000 µM	48 h/5 days	AChE activity and generation of ROS	Sub-lethal concentrations of CPF potentiated glutamate toxicity, but glutamate-induced ROS production was not affected. CPF toxicity was not well correlated with AChE inhibition	Amani et al. (2016) http://dx.doi.org/10.22074/CELLJ.2016.4575
Cerebellar granule neurons from PND 7 mice	0–100 µM	60 min/24 h	ROS, lipid peroxidation, GSH levels, GSSG/GSH ratio	CPF increased intracellular levels of ROS and caused lipid peroxidation	Giordano et al. (2007) http://dx.doi.org/10.1016/j.taap.2006.09.016
Rat cerebellar granule cells	0–200 µM	6 days	Modulation of neurodevelopmental gene expression	Neurofilament genes: concentration-dependent decreases in the expression of *Nefh* (DIV 4 and 7) and *Nefl* (DIV 4) Synaptic genes: significant reductions in the expression of *Syp* (DIV 7) and *Gabrd* (DIV 4 and 7)	Padhi et al. (2022) http://dx.doi.org/10.1016/j.yrtph.2022.105211
Cerebral cortices from rat embryonic day 17–18 rats	0.001–10 µM	24 h	Axonal transport of membrane-bound organelles	Decrease in the velocity and percentage of transport of membrane-bound organelles moving in the anterograde direction, an increase in the number of stationary membrane-bound organelles, and a decreased frequency in the movement of MBOs associated with CPF	Gao et al. (2017) http://dx.doi.org/10.1016/j.neuro.2017.06.003
Primary rat astrocytes from foetal day 21	0–150 µM	24 h/48 h	DNA synthesis	CPF and CPO caused a concentration-dependant inhibition of [^3^H]-thymidine incorporation in primary rat astrocytes	Guizetti et al. (2005) http://dx.doi.org/10.1016/j.tox.2005.07.004
Sympathetic neurons of perinatal rats	0.0001 µM–10 µM	24 h/72 h	Axonal and dendritic growth	CPF did not change the number of axons extended per neuron but caused a concentration-dependent decrease in total axonal length per neuron. CPF caused a significant increase in total dendritic length per neuron	Howard et al. (2005) http://dx.doi.org/10.1016/j.taap.2004.12.008
Foetal rat telencephalon cells, both immature (DIV 5–15) and differentiated (DIV 25–35)	0.01–100 µM	10 days	Cell maturation (neuronal and glial markers)	In immature cultures, CPF caused a significant decrease in acetylcholine transferase activity, while it did not have a significant effect in differentiated cultures. No effect on glial markers in immature nor in differentiated cultures	Monnet-Tschudi et al. (2000) http://dx.doi.org/10.1006/taap.2000.8934
Foetal rat telencephalon cells	0–1 µM	10 days (DIV 5–15)	Glial neurotoxicity	Glial cells provide neuroprotection against CPF toxicity	Zurich et al. (2004) http://dx.doi.org/10.1016/j.taap.2004.05.003
Rat C6 glioma cells	10 µM	24 h	Transglutaminase activity	CPF caused an increase in transglutaminase activity	Muñoz et al. (2010) http://dx.doi.org/10.1016/j.tiv.2010.07.011
Rat C6 glioma cells	0–10 µM	4 h	Effect on microtubule proteins	CPF decreased the levels of the cytoskeletal protein MAP1B, whereas the levels of the cytoskeletal protein tubulin and MAP2c were not significantly affected	Sachana et al. (2008) http://dx.doi.org/10.1016/j.tiv.2008.02.022
Rat C6 glioma cells	0.2–50 µg/mL	2 h/24 h	DNA synthesis, ROS, cell differentiation, nuclear transcription factor DNA-binding activity	In undifferentiated cells, CPF selectively inhibited DNA synthesis at non-cytotoxic doses. In differentiating cells, CPF elicited changes such as inhibition of DNA synthesis, impairment of G-function within the AC signalling cascade, impairment of cell differentiation, disruption of nuclear transcription factor DNA binding activity and production of ROS	Garcia et al. (2001) http://dx.doi.org/10.1016/S0006-8993(00)03189-9
Rat hippocampal cells	0–100 µM	24 h/48 h/72 h	Oxidative stress	CPF led to a significant decrease in the survival of hippocampal neurons	Salyha (2013) http://dx.doi.org/10.1007/s11062-013-9356-7
Primary cultures of rat embryonic cortical and hippocampal neurons (ED18); primary cultures rat astrocytes (1-day-old)	0.001, 0.01, 0.1, 1, 10 µM	1 h	Cell viability; AChE activity;	CPF increased pCREB expression (but not total CREB) in cortical and hippocampal neurons (but not astrocytes) in conditions where AChE activity was inhibited by 25%	Schuh et al. (2002) http://dx.doi.org/10.1006/taap.2002.9445
Primary cultures of rat dorsal root ganglia neurons from ED 15	0–10 µM	24 h	Inhibition of axonal growth	CPF altered morphogenic events critical to establishing neuronal connectivity, in part by interfering with the morphogenic activity of AChE Morphometric analyses indicated that CPF did not alter the number of extended axons, but did alter axonal length at non-cytotoxic and non-AChE inhibiting exposures	Yang et al. (2008) http://dx.doi.org/10.1016/j.taap.2007.11.005

^a^This study is mixed human + rodent; therefore, it is also mentioned in Table 2

#### PC12 cells

PC12 cells are derived from rat adrenal pheochromocytoma cells. When exposed to nanomolar concentrations of nerve growth factor (NGF), PC12 cells changes into an electrically excitable and mature cellular state that resembles sympathetic neurons (Fujita et al. 1989). NGF induces the growth of neurites in PC12 till 1–2 µm in width and more than 100 µm in length (Liebetrau et al. 2003).

The effect of CPF on PC12 has been well characterised in a number of studies. These in vitro studies have shown that CPF alters neurodifferentiation by inhibiting NGF-induced neurite outgrowth and causing a range of molecular changes such as impairment of neurotransmitter and neurotrophic homeostasis and adenylyl cyclase (AC) signalling, induction of oxidative stress and a variety of other effects including alteration of protein kinase C, cell cycle, apoptosis and others.

##### Inhibition of neurite outgrowth

Only one study investigated the effect of CPF on neurite outgrowth; after exposing PC12 cells to CPF for 5 days, a dose-dependent inhibition of NGF-induced neurite outgrowth was observed. The effect was not significant at 9 µM, but reached statistical significance at 29 µM, and neurite outgrowth was completely suppressed at 171 µM (Christen et al. 2017).

##### Neurotransmitter homeostasis

Several studies have reported effects of CPF on neurotransmitter homeostasis in PC12 cells. Exposure of undifferentiated cells to 5 µM CPF for 3 days induced a reduction in DNA synthesis without reducing viability; a small but significant increase in the expression of the enzymatic marker of the catecholamine phenotype, TH, was observed without affecting the expression of the enzymatic marker of the cholinergic phenotype, choline acetyltransferase (Jameson et al. 2006). However, when CPF was added to the culture at the same time as NGF, inducing differentiation, after 7 days of exposure to 5 µM, the pesticide significantly reduced choline acetyltransferase but not TH activity, whereas when CPF was added to the culture after 4 days of treatment with NGF, TH was slightly increased without affecting choline acetyltransferase (Jameson et al. 2006), suggesting an alteration of the phenotypic fate of neuronal precursors by CPF.

Generally, inhibition of AChE results in cholinergic hyperstimulation and acute nerve toxicity. However, AChE has also non-enzymatic functions related to neurodevelopment and repair of neurological damage. AChE has two known variants, the rare form (AChE-R), which is preferentially induced after injury and is involved in repair and protective functions, and the synaptic form (AChE-S), which enhances neurotoxicity. Exposure of PC12 cells to 30 µM CPF for 48 h in the presence of NGF increased the expression of both AChE-R and AChE-S isoform genes by approximately 20%. However, the same exposure to CPO (the active metabolite of CPF and a much more potent AChE inhibitor) increased the expression of the gene encoding the AChE-R form by 20% and did not affect the gene encoding the AChE-S isoform (Jameson et al. 2007). Taken together, these results support the non-enzymatic functions of AChE as involved in the neurodevelopmental impairments induced by CPF.

Slotkin and Seidler (2008) used PC12 cells to study the effect of CPF on serotonin (5HT) homeostasis. Exposure of PC12 cells (both undifferentiated and in the presence of NGF) to 30 µM CPF for up to 72 h induced upregulation of the tryptophan hydroxylase gene (which encodes the rate-limiting enzyme in the synthesis of 5HT). The same exposure caused a downregulation of the expression of the gene encoding the presynaptic high affinity 5HT transporter (*Slc6a4*) in both (differentiated and undifferentiated) cultures. As regards vesicular monoamine transporter genes, a small reduction in the expression of *Slc18a1* was observed only in differentiating cells, without significant changes in undifferentiated cells. However, the effect on *Slc18a2* expression was more robust, with stimulation during differentiation and repression in undifferentiated cells. No significant changes in the expression of monoamine oxidases a and b were reported. In both differentiating and undifferentiating cell cultures, the expression of the genes encoding the 5HT receptor subunits *Htr1a*, *Htr1d*, *Htr2b* and *Htr5a* was significantly increased and that of *Htr3b* decreased. The effects of CPF on the expression of the genes encoding the 5HT receptor subunits *Htr1b* and *Htr6* were more selective (upregulated only in differentiating cells), *Htr5b* (upregulated only in undifferentiated cells) and *Htr7* (downregulation restricted to differentiating cells). Overall, this study provides evidence linking the neurodevelopmental toxicity of CPF to alterations in the 5HT pathway.

The homeostasis of the neurotransmitter glutamate is also affected by CPF. Exposure of PC12 cells to 30 µM CPF for 24–72 h induced several changes in the transcriptome. CPF did not significantly change the expression of glutamate receptor genes (*Gria1*, *Gria3* and *Grik3*) between undifferentiated and differentiated cells (16–18% upregulation), while for *Gria2*, *Gria4* and *Grin2a* downregulations between 30 and 50% were observed. However, the upregulation of *Grid2*, *Grin1* and *Grin2b* and the downregulation of *Grin3b* were restricted to differentiating cells. CPF exposure did not alter the expression of genes encoding metabotropic glutamate receptors in undifferentiated cells. However, differentiating cells showed increased expression of *Grm4* (up to about 20%) and decreased expression of *Grm5* (up to 50%) and *Grm6* (up to 30%) (Slotkin and Seidler 2009a). Taken together, this work suggests that excitotoxicity is an underlying mechanism of the developmental toxicity of CPF.

In a follow-up study to clarify the effects on glutamate transporters, PC12 cells (both in the presence and absence of NGF) were exposed to 30 µM CPF for 24–72 h than performing microarray analysis (Slotkin et al. 2010). In undifferentiated cells, a transient and robust increase in the expression of solute carrier family 1 (glial high affinity glutamate transporter) member 3, and smaller but more sustained increases in the expression of members 4 and 5 of the same family were observed, whereas the expression of the gene encoding vesicular glutamate transporter 3 was reduced. In differentiating cells, CPF induced a global upregulation of the eight glutamate transporters after 24 h of exposure. Overall, this study further supports excitotoxicity as a significant contributor to the neurodevelopmental impairments caused by CPF.

In same treatment conditions, another microarray analysis in both differentiated and undifferentiated PC12 cells evidenced that CPF induced upregulation of both light and medium neurofilament polypeptides, while growth-associated protein 43 and heavy neurofilament polypeptide remained unchanged (Slotkin and Seidler 2009b).

The homeostasis of ACh-related genes was also altered by CPF at 30 µM (Slotkin and Seidler 2009b) by strongly suppressing the expression of choline acetyltransferase and the high-affinity presynaptic choline transporter in PC12, in both differentiated and undifferentiated states. The expression of the low-affinity choline transporter, which also transports creatine, the vesicular ACh transporter and AChE, were upregulated to a small but significant extent by CPF, while butyrylcholinesterase was strongly downregulated in undifferentiated cells. CPF induced different changes in the expression of muscarinic ACh receptors, as subtype 1 was strongly downregulated in undifferentiated cells and upregulated in cells exposed to NGF. On the contrary, CPF induced an upregulation of subtype 2 and a downregulation of subtype 3, irrespective of the differentiation state. Finally, CPF induced a significant reduction in the expression of ACh muscarinic receptor subtype 5 only during differentiation, and major changes in the expression of certain nicotinic ACh receptor subunits according to the differentiation state. In undifferentiated cells, CPF downregulated nicotinic ACh receptor subunit 2 and upregulated subunits 4 and 10. In differentiating cells, CPF induced a small downregulation of nicotinic ACh receptor subunit 5, a substantial increase in nicotinic subunit 10 and a time-dependent change in subunit 9. A strong upregulation of nicotinic ACh receptor β subunit 3 and a variable effect on nicotinic ACh receptor ε subunit (downregulation in undifferentiated cells and upregulation after 24-h exposure in cells undergoing differentiation) were also observed.

Exposure to CPF 30 µM also induced a significant increase in the expression of TH and dopamine β-hydroxylase (both genes directly involved in catecholamine synthesis). The same effect was observed for the presynaptic high-affinity noradrenaline transporter. A small reduction of the vesicular monoamine transporter in differentiating cells and a strong suppression of the high-affinity dopamine transporter in undifferentiated cells was also recorded. CPF exposure induced an upregulation of dopamine receptor subtype 1a independent of the differentiation state. Similarly, dopamine receptor subtype 3 showed small increases in response to CPF in undifferentiated but not in PC12 cells under differentiation (Slotkin and Seidler 2009b).

Overall, these studies evidenced a major impact of CPF on choline-related genes as well as muscarinic, nicotinic and dopamine receptors, with potential consequences on the homeostasis of the corresponding neurotransmitters. Indeed, CPF affected the expression of genes related to neuronal growth and extension in PC12 cells and promoted differentiation into cells with a dopamine phenotype at the expense of the ACh phenotype (Slotkin and Seidler 2009b). However, Slotkin and co-workers (2012) conducted experiments demonstrating that glucocorticoid dexamethasone was able to revert this dopamine phenotype promotion induced by CPF, enhancing the cholinergic phenotype.

##### Neurotrophic factors homeostasis

Slotkin and Seidler (2010a) exposed PC12 cell cultures (with and without NGF) to 30 µM CPF for 24–72 h and used microarrays to assess the expression of 40 genes encoding neurotrophic factors and circuitry. In undifferentiated PC12 cells, the transcription of neuropeptide Y, neurotensin and pre-proenkephalin was significantly upregulated, whereas the transcription of cholecystokinin, prodynorphin, somatostatin and tachykinin 1 was downregulated. The following receptors were induced by CPF in undifferentiated cells: opioid δ1, oxytocin, tachykinin 3 and vasoactive intestinal peptide 1, whereas the expression of mRNA encoding arginine vasopressin receptor 1a and somatostatin 2 was decreased. The transcriptome of differentiated cells was affected to a greater extent with significant increases in the expression of cholecystokinin, corticotropin-releasing hormone, neuropeptide Y and neurotensin, with smaller increases in the transcription of galanin, pre-proenkephalin and tachykinin 1. Only tachykinin 4 showed a downregulation. CPF had no significant effect on the expression of neuropeptide receptors in differentiating cells. In conclusion, alterations in the gene expression of several neuropeptides and their receptors are another underlying molecular mechanism of CPF-induced neurodevelopmental toxicity.

In another toxicogenomic study (Slotkin et al. 2008), PC12 cells exposed to 30 µM CPF for 24 and 72 h displayed, in the undifferentiated state, an upregulation of the neurotrophic tyrosine kinase receptor 1 by about 10%, while the isoform 3 was downregulated by 60% after 24 h of exposure. In these undifferentiated cells, expression of *Bdnf* and *Ngf γ* was downregulated by 20 and 60%, respectively. For fibroblast growth factors (Fgfs), the only statistically significant changes in expression were downregulations of factors 18 (by 20%); 20 (by 70% after 24 h of exposure and by 40% after 72 h of exposure) and 23 (by 40%). Regarding neurotrophic factors and their receptors, only neurotrophic tyrosine kinase receptor 1 and *Ngf γ* had a similar expression between undifferentiated and differentiating cells, while the expression of neurotrophic tyrosine kinase receptor 3 was downregulated in differentiated cells, but to a lesser extent (by about 40%) than in undifferentiated cells. In contrast to undifferentiated cells, the expression of neurotrophic factors 3 and 5 was downregulated in differentiated cells (by 40% for isoform 3 and by 35% for isoform 5), while the expression of *Bdnf* was upregulated by 40% after 24 h of exposure. *Fgf17* and *Fgfr2* expression was altered in differentiating cells but not in undifferentiated cultures. *Fgf20* was downregulated in differentiated cells, but to a lesser extent than in undifferentiated cells. Overall, this study suggests that CPF alters the expression of critical neurotrophic factors and their receptors, as well as signalling pathways that control neuronal cell differentiation.

##### Oxidative stress

Early studies showed that CPF has the ability to induce acute (but not chronic) increases in ROS. Ten-minute exposure of PC12 cells resulted in statistically significant increases in ROS production at 1.4, 5.6 and 140 µM (Crumpton et al. 2000a). Exposure to CPF (but not CPO) statistically increased ROS levels in undifferentiated cells exposed for 10 min but not at 24 or 48 h, while exposure for 72 h in conditions that inhibit neurite outgrowth did not significantly alter ROS production (Crumpton et al. 2000a).

Lassiter and co-workers (2009) found that exposure of undifferentiated PC12 cells to 30 µM CPF for 24 h increased malondialdehyde formation by approximately 20%, which instead was not significantly increased in cell cultures undergoing neurodifferentiation exposed to the same concentration of CPF for 4 days.

In other studies, exposure of PC12 cells undergoing differentiation to CPF induced an increase in ROS production, with undifferentiated PC12 cells appearing more sensitive to oxidative stress, as described above. Exposure of undifferentiated PC12 cells to 100 µM CPF for 24 h increased lipid peroxidation by approximately 80%, whereas in cells previously differentiated, then exposed to CPF for 8 days, the increase was only 47% (Qiao et al. 2005). At 14 µM CPF, the percentage increase in lipid peroxidation in cells undifferentiated for 8 days and differentiated for 8 days was approximately 30 and 21%, respectively, after 24-h exposure (Qiao et al. 2005). In another study, 30 and 50 µM CPF induced a modest (about 10%) increase in lipid peroxidation in differentiating PC12 cells after 6 days of exposure (Slotkin and Seidler 2010b).

In PC12 undifferentiated cells, three superoxide dismutase genes were modestly (about 20%) but significantly downregulated after exposure to 30 µM CPF, while catalase expression was not affected. Two of the six glutathione peroxidase genes were significantly upregulated (up to about 20%) in undifferentiated PC12 cells after exposure to 30 µM CPF. Transcription of the genes encoding glutathione S-transferases in undifferentiated cells showed statistically significant increases in response to CPF exposure, but the magnitude of the effect did not exceed 20%. In general, the response of antioxidant genes to CPF was greater in cells undergoing neurodifferentiation than in undifferentiated cells. 30 µM CPF induced a significant upregulation (20%) of genes encoding catalase and glutathione synthetase in differentiated PC12 cells. The transcription of ten glutathione S-transferase genes in PC12 cells cultured in the presence of CPF and NGF was significantly increased (between 20 and 40%), while three of these glutathione synthetases were moderately downregulated (always below 10%) (Slotkin and Seidler 2009a).

Overall, these studies demonstrated that the exposure to CPF causes an increase in oxidative stress, especially in undifferentiated cells, whereas the increase in the mechanism of antioxidant defences was more pronounced in differentiated cells.

##### AC signalling

Slotkin and co-workers (2007a) exposed PC12 cells undergoing neurodifferentiation to 30 µM CPF for 6 days. They found a reduction of about 30% in basal AC activity induced by CPF.

As a confirmation, in another study (Garcia et al. 2001), exposure of PC12 cells (both undifferentiated and undergoing neurodifferentiation) to 50 µM CPF for 2 and 6 days did not affect AC activity in undifferentiated PC12 cells; however, in differentiating cells exposed to CPF during the first 2 days of differentiation, AC activity was reduced by about 10%, whereas when the exposure was extended to 6 days, the inhibition of AC activity was lower (about 5%). In conclusion, these studies demonstrated a clear effect on AC signalling by CPF exposure; in addition, exposure during early differentiation reprogrammed AC signalling cascades, which may be a relevant mechanism of non-cholinergic toxicity.

##### Alterations in DNA synthesis

There are several studies in the literature reporting CPF ability to inhibit DNA synthesis. For example, exposure of undifferentiated PC12 cells to 30 µM CPF for 1 or 24 h reduced DNA synthesis by 21 and 13%, respectively (Lassiter et al. 2009). However, in PC12 cells undergoing neurodifferentiation, this inhibition did not appear to be time-dependent, as exposure to 30 µM CPF for 6 days caused only 17% inhibition, although this was accompanied by a 14% increase in the total protein/DNA ratio and no change in the ratio of membrane protein to total protein (Lassiter et al. 2009).

The effects of CPF on DNA synthesis were also confirmed in another study where CPF caused a modest (6–19%) but significant concentration-dependent and time-independent inhibition of DNA synthesis in undifferentiated PC12 cells after exposure to 30 or 50 µM CPF for 1, 4, 24 h or 6 days (Slotkin et al. 2007b). The onset of differentiation dramatically reduced absolute DNA synthesis, which, however, was also inhibited to a similar extent in undifferentiated cells (Slotkin et al. 2007b). Total protein/DNA and membrane/total protein ratios were significantly increased in differentiated (approximately 22% and 8%, respectively) and undifferentiated (approximately 5% and 9%, respectively) cell cultures (Slotkin et al. 2007b). These results were confirmed in another study from the same laboratory focussing on the effects of CPF on AC signalling (Slotkin et al. 2007b).

Finally, another study also reported that total DNA/dish was moderately reduced in differentiated PC12 cells after exposure for 6 days to 30 µM CPF (reduction of about 5%) or 50 µM (reduction of about 13%) (Slotkin and Seidler 2010b). As in other cases, this reduction was accompanied by a 5–14% increase in the total protein/DNA ratio (Slotkin and Seidler 2010b).

Overall, these studies suggest that in differentiated cells, the decrease in DNA synthesis is higher compared to undifferentiated cells, with unavoidable consequences on the protein/DNA ratio and on cell functionality.

##### Other molecular alterations related to neurodifferentiation

In one study, undifferentiated PC12 cells exposed to 50 µg/ml (approximately 154 µM) CPF for 48 h displayed a reduced expression of *Sp1* (a transcription factor involved in the expression of genes during early developmental stages) by about 40%, while the expression of *Ap-1* a heterodimeric transcription factor involved in the regulation of differentiation, proliferation and apoptosis) was reduced by about 10%, but not significantly. However, in same cells undergoing neurodifferentiation, the expression of *Ap-1* was statistically reduced by 30% and the reduction in the expression of *Sp1* (by about 20%) was not significant (Crumpton et al. 2000b). Overall, this study shows that CPF affects neurodevelopment in part by altering the activity of transcription factors involved in the basic machinery of cell replication and differentiation.

Slotkin and Seidler (2009c) exposed PC12 cell cultures to 30 µM CPF for 24 or 72 h and then used microarrays to determine the effect of exposure on the expression of 11 different protein kinase C isoforms and 3 regulatory binding proteins. In undifferentiated cells, CPF exposure upregulated three protein kinase C isoforms (*prkcn*, *prkce* and *prkcb1*) and the regulatory binding protein *prkcdbp*. Only two protein kinase C isoforms (*prkcm* and *prkci*) were downregulated. A net upregulation of the *prkca* and *prkci* isoforms and the regulatory protein *prkcdbp* was reported in cells cultured in the presence of NGF. However, *prkcm*, *prkc3*, *prkcq* and *prkcn* showed a transient (statistically significant only after 24 h of exposure) upregulation with a return to basal or slightly reduced expression levels by 72 h of exposure. The opposite was seen for *prkcb1*, which was downregulated at 24 h and upregulated at 72 h. In conclusion, according to these authors, alterations in the regulation of protein kinase C pathways may be involved in the neurodevelopmental toxicity induced by CPF.

In another transcriptomic study (Slotkin and Seidler 2011), no significant changes were observed in the expression of 21 genes related to Parkinson’s disease in PC12 cells (either undifferentiated or neurodifferentiated) exposed to 30 µM CPF for 24 h. However, after 72 h of exposure, two genes were strongly upregulated (α-synuclein and 2-amino-3-carboxymuconate-6-semialdehyde decarboxylase) and four others were significantly downregulated (ATPase type 13A, glucosidase, beta, acid 3 (cytosolic), bone marrow stromal cell antigen 1 and lysosomal-associated membrane protein 3). Thus, this study provides some mechanistic links between CPF exposure and Parkinson’s disease.

Exposure of PC12 cell cultures to 30 µM CPF altered the expression of 32 and 41% of genes involved in the cell cycle in undifferentiated and differentiating cells, respectively. The percentage of apoptosis-related genes with altered expression was 33 and 38% in undifferentiated and differentiating cells, respectively (Slotkin and Seidler 2012). Overall, this study demonstrated that cell cycle and apoptosis are targeted by CPF.

#### Embryonic stem cells

Visan and co-workers (2012) studied the differentiation of D3 mouse embryonic stem cells into neural cell types by monitoring the expression of Map-2, a marker of neural growth, axonal regeneration and synaptic plasticity. CPF inhibited the formation of Map-2-positive cells in a concentration-dependent manner with an IC_50_ of 4 µM. However, this process occurred at cytotoxic concentrations, while undifferentiating cells were more resistant to CPF.

In a similar study on D3 mouse embryonic stem cells exposed for 12 h–10 µM CPF (non-cytotoxic exposure conditions), the gene expression of *Flk1* (marker of mesoderm), *Oct4* (marker of pluripotency), and *AChE* (marker of ectoderm) were statistically reduced by 40%, 80%, and 80%, respectively, while the expression of *Pnpla6* (marker of mesoderm) was increased by 70% (Estevan et al. 2013).

1 µM CPF suppressed the expression of the glial phenotype in differentiating neural stem cells established from mice at embryonic day (ED) 14, while the neuronal phenotype was enhanced up to 20 µM CPF (Slotkin et al. 2016). This combination of effects (reduction of the glial phenotype and enhancement of the neuronal phenotype) resulted in a significant decrease in the glia/neuron ratio.

Overall, these studies evidenced the sensitivity of embryonic stem cells to CPF exposure, with severe impairment of the expression of genes regulating embryo patterning and imbalance of glia/neuro phenotypes.

#### Rat embryonic mesencephalic dopaminergic neurons

The effects of CPF on N27 rat embryonic mesencephalic dopaminergic neural cells were investigated by Singh and co-workers (2018). CPF caused a dose-dependent reduction in N27 cell viability by approximately 50% and 70% after 24 h of exposure at 50 and 100 µM, respectively. Significant DNA fragmentation was also reported for exposures at 100 and 300 µM. Exposure to 10 µM CPF induced time-dependent activation of caspase 3, PARP cleavage, ROS and mitochondrial superoxide levels in N27 cells, and decreased GSH and MMP levels. CPF (10 µM) also inhibited the expression of the anti-apoptotic protein Bcl-2 and increased the recruitment of STAT1 to the endogenous NOX-1 promoter. A reduction in basal respiration and ATP production were also observed with a concurrent increase in the release of cytochrome c from mitochondria into the cytosol. CPF also induced autophagy as evidenced by increased expression of LC3B, Beclin 1 and p62. Collectively, these findings suggest that CPF may potentially cause dopaminergic neurotoxicity with associated neurobehavioural deficits and neurodegenerative diseases triggered by apoptosis or autophagy.

#### Cortical neurons

CPF significantly inhibited the mean firing rate (number of spikes), mean burst rate (number of bursts/min), mean burst duration and percentage of spikes in burst in cortical neurons obtained from 18-day-old Wistar rats in a concentration-dependent manner, with IC_50_ values for 20-min exposure equal to 21.5 µM, 21 µM, 34.9 µM and 24.8 µM, respectively; while at 100 µM, CPF induced an almost complete block of spontaneous neuronal electrical activity (Alloisio et al. 2015). Another study on same cells established from rats at ED 17 or at birth (Caughlan et al. 2004) found an increase in apoptosis from a basal level of 30% to 59% at 24 h and 76% at 72 h after treatment with 50 µM CPF. This study also reported that embryonic neurons were more sensitive than postnatal neurons to CPF since an exposure to 30 µM of CPF did not induce apoptosis in the latter, while it caused a statistically significant increase in apoptosis in ED 17 cortical neurons.

Culture of cortical cells isolated from neonatal rats (postnatal day 0–1) at doses up to 10 µM CPF for 14 days inhibited the development of spontaneous electrical activity (Dingemans et al. 2016). A study in rat cortical cells from postnatal day 1 showed that 30 min exposure to CPF caused a concentration-dependent decrease in neuronal network activity, particularly in the number of spikes and network bursts. These effects also occurred at 24 and 48 h (van Melis et al. 2023).

Overall, these studies confirm the adverse effects of CPF on neuronal structure and integrity, also on cortical neurons, especially at the embryonic stage.

#### Cultured mouse and rat cerebellar neurons

Several studies have reported effects of CPF exposure on AChE activity, ROS generation, lipid peroxidation, GSH levels, GSSG/GSH ratio and modulation of neurodevelopmental gene expression in cultured cerebellar granule neurons.

A study by Amani and co-workers (2016) observed that sub-lethal concentrations of CPF enhanced glutamate toxicity in mouse cerebellar neurons established at PND 6–7, while glutamate-induced ROS production was unaffected. Thus, these authors suggested that CPF potentiates glutamate toxicity by mechanisms other than oxidative stress. Another study performed in cerebellar granule neurons of 7-day-old mice (Giordano et al. 2007) reported an increase in intracellular levels of ROS and lipid peroxidation at 1 µM CPF.

Modulation of neurodevelopmental gene expression was observed in differentiating rat cerebellar granule cells established at PND 8 by Padhi and co-workers (2022). No perturbation of gene expression was observed after exposure to 6.25 µM CPF, whereas at 12.5 and 25 µM CPF, concentration-dependent decreases in the expression of the neurofilament gene *Nefh* at DIV 4 and DIV 7 and for *Nefl* at DIV 4 were observed. The expression of the synaptic gene *Syp* was significantly reduced at DIV 7 following exposure to 12.5 and 25 µM CPF, while a significant decrease in *Gabrd* expression was observed at DIV 4 and DIV 7 following exposure to 25 µM CPF.

Exposure of mouse adipose tissue-derived stem cell neurons to 100 µM CPF upregulated the expression of neuronal marker genes such as *Nestin* (by 13-fold), *NeuN* (by 18-fold), *NEFL* (by fivefold) and *Syp* (by sixfold) at different time points in culture (Zarei et al. 2015). Immunohistochemistry also showed that CPF exposure was also able to reduce the number of cells expressing β-tubulin III and Map-2 proteins. Since these cells do not express AChE, the observed effects can be considered AChE-independent.

#### Cerebral cortices

Exposure of the cerebral cortices of ED 17–18 rats to CPF (Gao et al. 2017) decreased the velocity and percentage of movement of axonal transport moving membrane-bound organelles (MBO), increased the number of stationaries, and decreased the frequency of movement of MBOs associated with CPF. The impairment in axonal transport associated with CPF did not appear to be a result of its conversion to CPO. These effects may be significant given the importance of axonal transport in neuronal development and the function of fully developed neurons.

#### Glia cells

The effect on DNA synthesis was observed in primary rat astrocytes from foetal day 21 exposed to up to 150 µM CPF and CPO (Guizzetti et al. 2005) which caused a concentration-dependent inhibition of [^3^H]-thymidine incorporation from 10 µM.

Exposure to up to 10 µM CPF in rat C6 glioma cells under differentiation, induced by sodium butyric acid, showed an approximately 50% increase in transglutaminase activity compared to control (Muñoz et al. 2010) and a decrease in the levels of Map1b, while tubulin and Map2c were not significantly affected (Sachana et al. 2008). These results suggest that CPF may exert toxic effects directly on glial cell differentiation.

In undifferentiated C6 cells, CPF selectively inhibited DNA synthesis at non-cytotoxic doses, whereas in differentiating cells, CPF caused changes such as inhibition of DNA synthesis, impairment of cell differentiation, disruption of nuclear transcription factor DNA binding activity and production of ROS (García et al. 2001).

#### Sympathetic neurons

The study by Howard and co-workers (2005) with postmitotic sympathetic neurons dissociated from the superior cervical ganglia of perinatal rats (ED 20 to postnatal day 1) showed disruption of neuronal morphogenesis via opposing effects on axonal and dendritic growth. Axon outgrowth was significantly inhibited at concentrations ≥ 0.001 µM CPF, while a parallel enhanced BMP-induced dendritic growth was observed. CPF caused a concentration-dependent decrease in total axon length per neuron, with a threshold concentration of 0.001 µM. A statistically significant increase in total dendritic length per neuron, with a threshold concentration of 1 µM, was also recorded.

#### Telencephalic cells

In immature telencephalic cells obtained from 16-day foetal rats, CPF at 10 µM caused a significant decrease in ACh transferase activity, whereas it had no significant effect in differentiated cultures (Monnet-Tschudi et al. 2000). Comparison of the maturation-dependent effects of CPF on GABAergic neurons from 16-day foetal rats showed that there was no difference. Similarly, CPF had no effect on glial markers in either immature or differentiated cultures. In another study conducted between DIV 5 and 15, CPF induced a weak gliotic response at concentrations where neurons were already affected (Zurich et al. 2004).

#### Hippocampal cells

Some studies in hippocampal cells have investigated the effect of CPF on oxidative stress-induced death. Exposure to CPF at 1 µM resulted in a significant decrease in the survival of neurons from hippocampi of Wistar rat embryos (prenatal day 18) (Salyha 2013). In primary cultures of embryonic rat cortical and hippocampal neurons (ED 18) exposed to CPF, the expression of activated CREB (an intracellular protein that regulates the expression of genes that are important in dopaminergic neurons) increased in a dose–response manner, with IC_50_ of 0.06 nM and 1–10 nM, respectively. AChE activity was inhibited by 25% in cortical neurons treated with CPF at 1 and 10 µM (Schuh et al. 2002).

Exposure to CPF up to 10 µM altered morphogenic events in rat dorsal root ganglia neurons from ED 15, in part by interfering with the morphogenic activity of AChE. In addition, morphometric analyses showed that CPF altered axonal length at a threshold concentration of ≤ 0.001 µM without affecting cell viability (Yang et al. 2008).

#### Overall conclusions about rodent models

##### PC12 cells

The available database with PC12 cells shows that exposure of these cells to CPF induces a number of effects (Table 3). Some of the changes are clearly related to neurotoxicity, but others are not. Analysis of the published information suggests that exposure of PC12 cells undergoing neurodifferentiation causes a reduction in neurite outgrowth (Christen et al. 2017), a reduction in the cholinergic neural phenotype (Jameson et al. 2006), accompanied by an increase in the serotoninergic (Slotkin and Seidler 2008), dopaminergic (Slotkin and Seidler 2009b) and glutamatergic (Slotkin and Seidler 2009a; Slotkin et al. 2010) neural phenotypes, alterations in neuropeptide homeostasis (Slotkin and Seidler 2010a; Slotkin et al. 2008) and reductions in the expression of the transcription factor Ap-1 (Crumpton et al. 2000b), which is involved in the control of differentiation. All these myriad effects are responsible, to a greater or lesser extent, for the impairment of neurodifferentiation caused by CPF.

AC (Slotkin et al. 2007a; Adigun et al. 2010) and protein kinase C (Slotkin and Seidler 2009c) have also been proposed as targets for neurodevelopmental toxicity induced by CPF. These two enzymes are known to regulate many cellular processes by modulating second messenger cascades. It should be noted that the reported effects were found in both differentiating and undifferentiating cells and were generally modest, not markedly increased in differentiating cells as would be expected for a neurodevelopmental insult. Therefore, although it is plausible that part of the differentiation process may also be regulated by these two enzymes, the weight of evidence to date is not high enough to robustly conclude that AC and protein kinase C are targets of neurodevelopmental toxicity induced by CPF.

DNA synthesis alterations in PC12 cells exposed to CPF has been consistently reported in several studies (Lassiter et al. 2009; Slotkin et al. 2007b; Slotkin and Seidler 2010b). However, it should be noted that the reductions in DNA synthesis were always modest (always less than 20%), not time-dependent and usually not significantly higher in cells undergoing differentiation than in undifferentiated cells. Therefore, it is unclear whether this effect on DNA synthesis is related to the mechanisms of neurodevelopmental toxicity.

The analysis of the information also shows how the exposure to CPF evokes oxidative stress in PC12 cells (Crumpton et al. 2000a; Lassiter et al. 2009; Qiao et al. 2005) and increases antioxidative stress defences by increasing the expression of glutathione peroxidase, catalase and glutathione synthetase genes (Slotkin and Seidler 2009a). However, it should be noted that all these effects are generally of moderate magnitude and occur in both undifferentiated and differentiated cells. This suggests that oxidative stress may be an adverse effect of CPF exposure that targets general cell survival processes rather than a specific neurodevelopmental adverse effect.

In conclusion, the use of PC12 cells have provided a robust core of evidence suggesting that CPF is capable of altering neurodifferentiation mainly by impairing neurotransmitter and neuropeptide homeostasis.

##### Rodent embryonic cells

Three studies analysed the effects of CPF on the differentiation of different rodent embryonic cell lines (Table 3). One study reported a reduction in glial phenotype with a concomitant increase in neural phenotype in rat neural stem cells (Slotkin et al. 2016); this effect is opposite to the increase in gliogenesis reported in human neural stem cells (Sandoval et al. 2019). In contrast to the work of Slotkin (2016), another study in D3 mouse embryonic stem cells reported a reduction in neurogenesis, monitored by the reduction in the expression of Map-2-positive cells (Visan et al. 2012). However, this latter study reported this effect at cytotoxic concentrations that cause cytotoxicity, which does not occur in the neural phenotype in rat neural stem cells, suggesting that this reduction in neuronal differentiation may not be physiologically relevant. In addition, another study also reported changes in the expression of mesoderm, pluripotency and ectoderm lineages in D3 mouse embryonic stem cells at non-cytotoxic concentrations (Estevan et al. 2013). Overall, CPF is shown to be a potential developmental neurotoxicant in these rodent embryonic stem cells, as it is able to significantly alter the expression of several genes during in vitro differentiation.

##### Other neural rodent models

The study of CPF toxicity with NAMs based on different rodent models has generated a wide range of important information (Table 3). The most relevant effects caused by CPF were dopaminergic (Singh et al. 2018) and glutamate (Amani et al. 2016) neuronal toxicity, reductions in electrical neuronal activity (Alloisio et al. 2015; Dingemans et al. 2016), reductions in the expression of genes encoding neurofilament assembly and synapse formation (Padhi et al. 2022), oxidative stress (Giordano et al. 2007), reduction in axonal transport (Gao et al. 2017) and total axon length per neuron (Howard et al. 2005), alteration in glial differentiation (Sachana et al. 2008; Muñoz et al. 2010). Taken together, this information is consistent with the possibility that CPF is a neurotoxicant and neurodevelopmental toxicant acting at relatively advanced stages of development and also a general developmental toxicant acting at early stages of embryonic development.

### Avian models

Only two studies using avian models were considered eligible (Supplementary Information 1, Table 4). Recently, as part of Canada's Chemicals Management Plan, two different alternative avian test strategies were developed using pre-hatched avian embryos (Crump et al. 2021; Farhat et al. 2020), which are not regulated in the European Union, the US and Canada. One model used *Japanese quail* embryos (Farhat et al. 2020) and the other a wild species, double-crested cormorant eggs, collected from nests in an area of low background contamination (Crump et al. 2021). Table 4 summarises the different endpoints and main conclusions of these 2 studies.

**Table 4:** Comprehensive summary of adverse effects caused by CPF reported using avian models

Biological	CPF (exposure)				
Model	Dose	Time	Endpoint	Main conclusions	Doi link
*Japanese quail* embryos	0.56, 4.9, 41.1 µg/g egg	ED 0, 9, 16	Vitality and developmental abnormalities	CPF (41.1 μg/g) significantly increased developmental abnormalities and decreased embryo and gallbladder mass. No effect on survival up to the highest dose (41.1 µg/g egg). Club feet was the most common deformity observed	Farhat et al. (2020) http://dx.doi.org/10.1002/etc.4582
Double‐crested Cormorant eggs	0.04, 2.3, 25 µg/g egg	ED 0, 14, 26	Vitality and developmental abnormalities	No effects on survival or other apical outcomes up to the highest dose tested. Limited deformities observed	Crump et al. (2020) http://dx.doi.org/10.1002/etc.4922

Both were injected with chemicals, including CPF, at three different doses at ED 0. The embryos were observed at mid-term (ED 9 and 14 for *Japanese quail* embryos and double-crested cormorant eggs, respectively) and 2 days before hatching (ED 16 and 26, respectively).

CPF had no effect on survival of *Japanese quail* embryos at ED 9 up to 41.1 µg/g egg, but significantly reduced embryo mass at the same exposure. Such a high dose also induced an increase in gallbladder weight and severe malformations, including an increased incidence of clubfoot (5/9), lordosis (2/9), gastroschisis (2/9) and crossed beak (1/9) (Farhat et al. 2020).

CPF did not induce death or apical defects in double-crested cormorant eggs at doses of 0.04, 2.3 and 25 µg/g egg; in addition, very limited deformities were observed in double-crested cormorant embryos, such as bill deformities (2/21) in the low-dose group and anophthalmia (1/24) in the high-dose group (Crump et al. 2021).

In conclusion, Japanese quail embryos were more sensitive to CPF, showing reduced mass and malformations at high doses, whereas two-crested cormorant embryos showed limited deformities without significant apical defects or lethality. Despite focussing on apical endpoints, these models offer promising alternatives for assessing the environmental impact of CPF.

### Fish models

The most used fish model for developmental testing is zebrafish (8 reports), although two reports used larval lake sturgeon or rainbow trout embryos. Table 5 summarises the different endpoints and main conclusions of all these 10 studies.

**Table 5:** Comprehensive summary of adverse effects caused by CPF reported using fish models

Biological	CPF (exposure)				
model	Dose	Time	Endpoint	Main conclusions	Doi link
Zebrafish embryo	0.55, 1.66, 5.00 µM	24 hpf	Spontaneous tail coiling	Hyperactivity possibly related to AChE inhibition in zebrafish embryos	Ogungbemi et al. (2020) http://dx.doi.org/10.1016/j.ntt.2020.106918
Zebrafish embryo	750 and 3000 µg/L	96 hpf	Toxicity test, RNA sequencing, differential gene expression analysis, overrepresentation analysis, gene set enrichment analysis	No lethality. Enrichment of gene ontology terms associated with oxygen transport and response to decreased oxygen levels, muscle cell differentiation and muscle structure development. Desmin, telethionin and iodothyronine diiodinase 3b were upregulated	Reinwald et al. (2022) http://dx.doi.org/10.1016/j.chemosphere.2021.132746
Zebrafish embryo	0.001, 0.01, 0.1 and 1 µM	7 dpf	Behavioural analyses	Little to no movement by 7 dpf, with twitching behaviour and could not swim normally. Sub-chronic doses decreased thigmotaxis and swimming speed in zebrafish at 7 dpf. At 1 µM, altered body morphology	Richendrfer et al. (2012) http://dx.doi.org/10.1016/j.ntt.2012.04.010
Zebrafish embryo	200 and 400 µg/L	72 hpf	qPCR, AChE activity, CAT and SOD activities, glutathione concentration, developmental toxicity	AChE activity inhibited at 400 µg/L AChE gene expression: increased at 200 µg/L and reduced at 400 µg/L CAT, SOD, GPx activities and total glutathione were not affected. Limited effects of CPF on apical endpoints (embryo mortality, pericardial area, and heartbeat frequency)	Rodríguez-Fuentes et al. (2015) http://dx.doi.org/10.1016/j.cbpc.2015.04.003
Zebrafish Tuebingen embryos	10, 1, 0.1, 0.01, 1e^−03^, 1e^−04^, 1e^−05^ µM	8–32 h hpf	Embryo toxicity test	Activation of AhR leading to dysfunction of cardiovascular system with consequent developmental defects and death	Gou et al. (2023) http://dx.doi.org/10.1016/j.jhazmat.2023.130958
Larval Lake Sturgeon	5, 500 and 2000 ng/L	21, 36 and 67 dph	Thyroid follicular development	At 6 dph, follicular cell height was the greatest in larvae treated with the lowest dose. At 9 dph, the follicular cell height was higher in all the treatment groups. At 12 dph, follicular cell height was highest in the medium treatment group. At 21 dph, follicle cell nucleus height was significantly lower in the medium treatment	Brandt et al. (2015) http://dx.doi.org/10.1016/j.chemosphere.2015.03.031
Rainbow trout embryos and larvae	0.3 and 3 μg/L	21 dph	Embryonic and larval survival; hatching rate; photomotor response; AChE activity; genotoxicity; lipid peroxidation and protein carbonyls; gene expression	The hatching rate was decreased after 7 and 8 days but not at the 9th. No significant developmental alterations. Larvae exposed to 3 µg/L covered a shorter distance and were less mobile with changing light conditions. The expression of oestrogen receptor beta gene was downregulated, whereas *cyp19a1* gene was repressed only by the higher dose	Santos et al. (2021) http://dx.doi.org/10.1002/etc.5183
Zebrafish embryos and larvae	6.25, 10 and 25 mg/l for embryos; 0.1875, 0.375 and 0.75 mg/l for larvae	24, 96, 120 and 144 hpf	Embryotoxicity, teratogenesis, spontaneous tail coiling, locomotor activity	At 24 hpf, CPF 25 mg/L determined 20% lethality in embryos, the remaining individuals exhibiting pericardial and yolk sac oedema. At 144 hpf, the lowest concentration (6.25 mg/L) induced 65% lethality. Skeletal deformities and sidewise position of larvae were observed in the 1 mg/L treated group. Frequency and total duration of spontaneous tail coilings increased in CPF-treated embryos at 24–26 hpf. The mean turn angle was increased by CPF mainly at 96 hpf; the total duration of movement increased at 96 hpf	Selderslaghs et al. (2010) http://dx.doi.org/10.1016/j.ntt.2010.03.002
Four lines of zebrafish embryos: wild-type, or transgenic for islet-1, NBT and Neurog-1	3 nM–1 µM	24 and 48 h	AChE activity, teratology screening, swimming behaviour, axon growth	Conversely to CPO, CPF did not inhibit AChE activity in zebrafish embryos, did not induce gross teratogenic effects and did not alter the swimming behaviour. Conversely to CPO, CPF had no effect on length and trajectory of GFP-positive axons in Neurog1 zebrafish and on axonal growth patterns in primary and secondary motoneurons in NBT or islet-1 zebrafish	Yang et al. (2011) http://dx.doi.org/10.1093/toxsci/kfr028
Zebrafish embryos	0.10, 0.25, 0.50, 0.75 and 1 mg/L		Embryo toxicity test, morphological observations, gene expression, apoptosis	The gene expression of vitellogenin was increased, whereas the expression of oestrogen receptor alpha was non-monotonically modulated. The hatching rate increased with the dose. At 5 dpf, spine deformation and cardiac oedema were observed in larvae, but with no significance. *C-myc* and cyclin D1 gene expression was upregulated. Small number of apoptotic cells, mainly around the heart area. *Bcl-2* and *bax* were, respectively, induced and repressed	Yu et al. (2015) http://dx.doi.org/10.1016/j.cbi.2015.06.010

#### Morphological developmental changes

In zebrafish embryos at 24 h post-fertilisation (hpf), CPF at 25 mg/l increased lethality by 20%, with survivors showing pericardial and yolk sac oedema. No effects were observed at lower concentrations. At 144 hpf, effects were also observed at lower doses; in particular, lethality was induced at 6.25 mg/L (65% of individuals), while skeletal deformities and larval lateralisation were observed at 1 mg/L (Selderslaghs et al. 2010). In other studies, CPF in the range of 0.01–1 µM or at 200–400 µg/L did not induce teratogenic effects in zebrafish embryos (Richendrfer et al. 2012; Yang et al. 2011).

Interestingly, CPF in the range of 0.1–1 mg/L increased the hatching rate of zebrafish embryos in a dose–response manner after 48 h of treatment, with approximately 100% survival in each group. However, at 5 dpf, signs of spinal deformation and cardiac oedema were observed at 0.75 and 1 mg/L CPF, although not significant. In addition, some apoptotic cells were detected around the heart area of zebrafish larvae treated with CPF at 0.75 and 1 mg/l for 48 h. Consistent with this finding, the ratio of *Bcl-2/Bax* gene expression increased, reaching a maximum at 0.5 mg/L, and then decreased at 0.75 and 1 mg/L (Yu et al. 2015).

CPF did not significantly affect the viability and hatching rate of rainbow trout embryos, although the percentage of hatched eggs exposed to CPF at 0.3 µg/L was significantly lower at days 7 and 8, but the effect disappeared from day 9. In addition, no significant developmental abnormalities were observed, except for slight and nonsignificant spinal deformities (Santos et al. 2021). CPF did not induce lethality in zebrafish embryos even at concentrations as low as 50 µM; however, developmental malformations such as yolk cyst, pericardial cyst, altered body axis, jaw, trunk and notochord were observed at this concentration (Gou et al. 2023).

Thyroid morphology was examined in lake sturgeon larvae following treatment with CPF; thyroid follicle cell height was increased at 6 and 9 days post-hatching (dph) only at the lowest dose of 5 ng/L, whereas at 12 dph, cell height was significantly increased by the intermediate dose (500 ng/L). Conversely, the same dose decreased thyroid follicle nucleus cell height in 21 dph larvae (Brandt et al. 2015). This suggests a potential endocrine disruption affecting thyroid system.

#### Neuromorphological changes

Effects on axon morphology were assessed in zebrafish embryos treated with CPF at 0.01–1 µM for 24/48 h. No effect on axon length or trajectory was observed in Neurog1 transgenic zebrafish (expressing GFP in Rohon-Beard and dorsal root ganglia sensory neurons and their axons), in contrast to CPO at 1 µM, which decreased axon length. Similarly, CPF did not affect axonal growth in either *NBT* or *islet-1* transgenic zebrafish lines (expressing GFP in primary and secondary motoneurons, respectively, and their axons), whereas CPO at 1 µM decreased the length of ventral and dorsal neurons in *NBT* zebrafish and at 0.1 µM in *islet-1* zebrafish (Yang et al. 2011).

#### AChE inhibition

Different results were obtained when assessing the inhibition of AChE activity. In zebrafish embryos treated with CPF in the range of 3 nM–1 µM for 24/48 h, no significant effect was observed compared to CPO, which decreased AChE activity (Yang et al. 2011). Similarly, AChE activity in rainbow trout larvae did not change significantly after exposure to CPF at 0.3 and 3 µg/L (Santos et al. 2021). In contrast, a statistically significant reduction in AChE was observed in zebrafish embryos treated with CPF at 400 µg/L for 72 h. Interestingly, *AChE* gene expression was increased by CPF at 200 µg/L (Rodriguez-Fuentes et al. 2015).

#### Neurobehavioural changes

The frequency and total duration of spontaneous tail coiling was increased by CPF in zebrafish embryos at 24–26 hpf with a dose–response profile, significant at 0.625, 2.5 and 10 mg/mL (Selderslaghs et al. 2010). The increase in spontaneous tail coiling frequency was also assessed after treating zebrafish embryos at 21 hpf with CPF in the range of 0.5–5 µM, with an EC_50_ of 1.85 µM (Ogungbemi et al. 2020). In terms of swimming behaviour, the mean turning angle was dose-dependently increased by CPF at 96, 120 and 144 hpf, being significant at 0.75 mg/L (at both 96 and 144 hpf) and 0.375 and 0.1875 mg/l (at 96 hpf only). At 96 hpf, CPF also induced a decrease in total exercise duration, especially at the two highest doses. At 120 hpf, no significance was observed, whereas at 144 hpf, the highest dose (0.75 mg/L) decreased the measured endpoint (Selderslaghs et al. 2010). In contrast to CPO, CPF did not affect touch-induced swimming behaviour (Yang et al. 2011). CPF at 0.1 and 0.01 µM significantly reduced thigmotaxis, the preference of larvae to remain at the edge of the well, in the absence of a visual stimulus; when exposed to a visual stimulus, only larvae treated with CPF at 0.1 µM reduced thigmotaxis. CPF at 0.1 and 0.01 µM significantly reduced also swimming speed, with or without visual stimulation (Richendrfer et al. 2012). A decrease in distance travelled and mobility was observed in rainbow trout larvae exposed to CPF at 3 µg/L, but only under changing light conditions (Santos et al. 2021).

#### Molecular mechanisms

The induction of genotoxicity, lipid peroxidation or protein carbonyl content was investigated in larvae or rainbow trout following exposure to CPF at 0.3 and 3 µg/L and no significant changes were observed (Santos et al. 2021). In addition, CPF at 200 and 400 µg/L did not affect CAT, SOD, GPx and total glutathione activity and/or gene expression in zebrafish embryos treated for 72 h compared to control embryos (Rodriguez-Fuentes et al. 2015).

Among the molecular endpoints, gene expression of c-myc and cyclinD1 (which regulate cell cycle and proliferation) was induced by CPF in zebrafish larvae, with non-monotonic responses in the range of 0.1–0.75 mg/L (Yu et al. 2015).

Two studies evaluated the endocrine effects of CPF; in zebrafish embryos, gene expression of vitellogenin was increased after treatment with CPF in a dose–response manner at 0.25, 0.5 and 0.75 mg/L; otherwise, the gene expression of the oestrogen receptor alpha (erα) was increased with a non-monotonic dose–response in the range 0.1–0.5 mg/L, being maximal at 0.25 mg/L and unaffected at 0.75 mg/l (Yu et al. 2015). In rainbow trout larvae, CPF suppressed gene expression of oestrogen receptor beta (erβ) at both 0.3 and 3 µg/L, and the gene encoding the aromatase enzyme (cyp19a1) only at 3 µg/L (Santos et al. 2021).

A transcriptomic study of zebrafish embryos treated at 750 or 3000 µg/L for 96 hpf revealed early gene expression changes at the low dose, which increased at the higher dose. GO terms associated with oxygen transport and response to reduced oxygen levels were significantly enriched in CPF-treated embryos at the higher dose. CPF also upregulated genes involved in cardiomyocyte development, including desmin (*desma*), telethonin (*tcap*) and genes encoding heat shock proteins (*hspb1* and *hspb8*). In addition, the GO terms related to cell–matrix adhesion and behaviour were enriched, the latter including the transcription factor npas4a, which is involved in brain development and synaptic plasticity, and iodothyronine deiodinase 3b (dio3b), an enzyme involved in thyroid hormone synthesis and thus highly relevant to brain development. CPF also induced the expression of ngf at the highest concentration (Reinwald et al. 2022).

A computational model calculating AOP-based scores, taking into account the starting points of the ToxCast and transcriptomics data, gave a high score that predicted the developmental toxicity of CPF well. The model also classified CPF into a cluster of chemicals with similar modes of action. In particular, activation of the Aryl Hydrocarbon Receptor (AhR) may be a crucial mechanism underlying AChE inhibition as well as the induction of cardiovascular defects (Gou et al. 2023).

#### Overall conclusions about fish models

In general, studies with fish models showed as CPF is able to induce general developmental impairments (e.g. pericardial and yolk sac oedema, cardiac apoptosis, or skeletal deformities) with exposures starting at around 1 mg/L (3 µM) (Selderslaghs et al. 2010; Gou et al. 2023; Yu et al. 2015). This concentration also correlates with AChE inhibition.

In addition, NAMs in other biological models indicates that CPF exerts neuromorphological changes during the development of neurons. While CPF did not significantly affect axon length trajectories, its active metabolite, CPO, exhibited notable axonal effects (Yang et al. 2011). This finding highlights the potential importance of biotransformation and route of exposure in the induction of adverse developmental effects. Indeed, oral exposure involves a relevant first-pass metabolism in the liver, which is the main organ involved in the biotransformation of CPF to CPO, whereas for exposures by other routes (e.g. inhalation or dermal), the first-pass metabolism in the liver may be less relevant due to a slower biotransformation of CPF. Furthermore, neurobehavioural changes induced by CPF exposure reported in in vivo rodent studies (López-Granero et al. 2016; Terry Jr. et al. 2012; Guardia-Escote 2018, Ribeiro et al. 2022, Berg et al. 2020, Biosca-Brull et al. 2022, Silva 2020) and in zebrafish, with alterations in the frequency and total duration of spontaneous tail coiling and swimming and thigmotaxic behaviour (Selderslaghs et al. 2010; Ogungbemi et al. 2020; Richendrfer et al. 2012), suggest that fish models are valid NAMs for studying the developmental toxicity of CPF.

### Mode of action of CPF for the induction of developmental toxicity and plausible AOPs

**Fig. 4:**
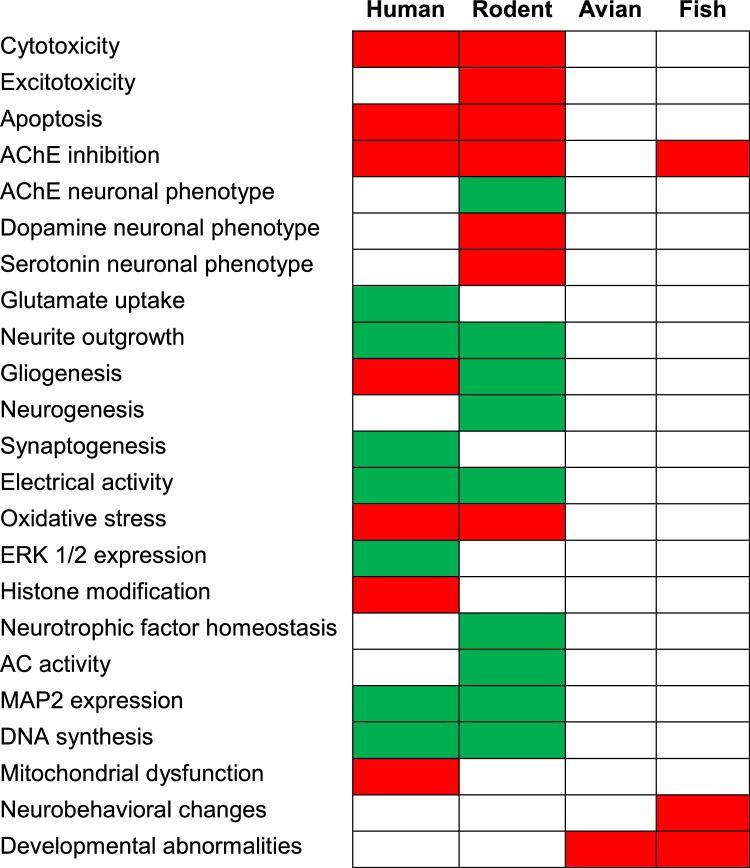
Heatmap panels summarising information on the adverse effects of CPF in several NAM models. Red = upregulation. Green = downregulation. Detailed information on each effect can be found in Tables 2, 3, 4 and 5

Having analysed all the available information in the 70 selected studies, and with the aim of gaining a mechanistic insight, we tried to determine whether the adverse effect of CPF was consistently repeated in the different models. To do this, we clustered all the endpoints more directly related to development in the semi-quantitative heat map shown in Fig. 4. The most recurrent adverse effect was AChE inhibition, which was found in three different models (human, rodent and fish). Among endpoints consistently found in two different models, there are increased cytotoxicity, apoptosis and oxidative stress reported in human and rodent NAMs, or reduction in neurite outgrowth, electrical activity, MAP2 expression and DNA synthesis also reported in human and rodent NAMs. The increase in developmental abnormalities was also reported in avian and fish NAMs. Only one of the developmental endpoints (gliogenesis) was reported inconsistently, being upregulated in human models, and downregulated in rodent models. In addition, a further set of 12 endpoints indicate either changes in gene expression (histone modification, AC activity and ERK 1/2 expression) during development, or nervous system dysfunction during development (increased excitotoxicity and downregulation of neurotrophic factor homeostasis and glutamate uptake) or effects leading to an abnormal developed nervous system such as downregulation of AChE neuronal phenotype, neurogenesis and synaptogenesis or upregulation of dopamine neuronal phenotype, serotonin neuronal phenotype and neurobehavioural changes (Fig. 4). Collectively, the evidence presented in Fig. 4, obtained exclusively through NAMs, suggests that it may be possible to determine the molecular mechanisms of the developmental toxicity of CPF reported in animal studies and the potential relevance to humans.

AOPs are conceptual frameworks that describe the sequence of biological events that lead to an AO in an organism or population as a result of exposure to a chemical. An AOP consists of a series of KEs that occur at different levels of biological organisation, from the molecular to the population level. The chain of events leading to an AOP is initiated by a MIE, which is the first interaction between a chemical and a molecular target in the organism. In the context of twenty-first century Toxicology, AOPs play a pivotal role in providing a mechanistic understanding of how chemicals can cause an AO in humans. This mechanistic approach is essential to overcome the limitations of traditional toxicity tests, which are often based on animal studies and may not be directly applicable to humans.

NAMs are essential for the development and validation of AOPs because they support the evidence of KEs in AOPs as well as the relevance of a given AO for humans, thus facilitating a more accurate risk assessment than that based on traditional methods. Information provided by NAMs identified in this review, may support the weight of evidence (WoE) of KEs belonging to one or more AOPs available in the Collaborative AOP Wiki (https://aopwiki.org/) (version 2.7). The alignment of NAMs to KEs also contribute to clarify the mode of action of CPF in inducing developmental effects and the relevance to humans. By gathering all the evidence described in the reported studies, we have found plausible associations of adverse developmental outcomes associated to CPF with up to 4 different AOPs (Table 6). All these four AOPs include *Homo sapiens* in their respective taxonomic applicability domain, and therefore, all the addressed AO are relevant for humans.

**Table 6:** AOPs with plausible association with adverse developmental effects associated to CPF

Type	Event ID	Title	Supported by:
AOP	405	Organophosphate chemicals induced inhibition of AChE leading to impaired cognitive function	
MIE	12	Acetylcholinesterase (AChE) Inhibition	✔ NT2 human cells Human iPSCs, NSC cells and 3D brain neurospheres ✔ N2a mouse cells ✔ Primary cultures of rat embryonic cortical, hippocampal neurons and astrocytes ✔ Zebrafish embryo
KE	10	Acetylcholine accumulation in synapses	✔ PC12^a^
KE	39	Increased cholinergic signaling	✔ PC12
KE	386	Decrease of neuronal network function	✔ Human NSC ✔ Foetal rat cortical neurons
AO	402	Cognitive function, decreased	• Animal experiments
AOP	475	Binding of chemicals to ionotropic glutamate receptors leads to impairment of learning and memory via loss of drebrin from dendritic spines of neurons	
MIE	875	Binding of agonist, Ionotropic glutamate receptors	✔ Human foetal astrocytes^a^
KE	388	Overactivation, NMDARs	✔ Huma foetal astrocytes^a^
KE	389	Increased, Intracellular Calcium overload	Data lacking in our study
KE	2078	Loss of drebrin	✔ Human NPC^a^ ✔ Rat C6 glioma cells^a^
KE	2242	Abnormality, dendritic spine morphology	✔ Human NPC ✔ Human NSC ✔ Human brain neurospheres ✔ Human LUHMES cells ✔ Mouse N2a neuroblastoma cells ✔ Rat adrenal pheochromocytoma PC 12 cells
KE	1944	Synaptic dysfunction	✔ Human NSC ✔ Rat cerebellar granule cells
KE	386	Decrease of neuronal network function	✔ Human NSC ✔ Rat foetal cortical neurons
AO	341	Impairment, Learning and memory	• Animal experiments
AOP	499	Activation of MEK-ERK1/2 leads to deficits in learning and cognition via disrupted neurotransmitter release	
MIE	2146	Activation of mitogen-activated protein kinase kinase, extracellular signal-regulated kinase 1/2	✔ Primary human astrocytes
KE	1339	Increase, intracellular calcium	Data lacking in our
KE	2151	Disruption, neurotransmitter release	✔ Rat adrenal pheochromocytoma PC 12 cells^a^
AO	341	Impairment, learning and memory	• Animal experiments
AOP	500	Activation of MEK-ERK1/2 leads to deficits in learning and cognition via ROS and apoptosis	
MIE	2146	Activation of mitogen-activated protein kinase kinase, extracellular signal-regulated kinase 1/2	✔ Primary human astrocytes
KE	1339	Increase, intracellular calcium	Data lacking in our study
KE	177	Mitochondrial dysfunction	✔ Human ReNCell NPC ✔ Cortical neurons from rat embryos
KE	1115	Increased, reactive oxygen species	✔ Human brain neurospheres ✔ Human LUHMES cells ✔ Rat adrenal pheochromocytoma PC 12 cells ✔ Mouse cultured cerebellar granule neurons ✔ Rat C6 glioma cells
KE	1262	Apoptosis	✔ Human ReNCell NPC ✔ Human LUHMES cells ✔ Rat foetal cortical neurons ✔ Zebrafish embryos
KE	352	n/a, neurodegeneration	✔ Human ReNCell NPC^a^ ✔ Human LUHMES cells^a^ ✔ Rat foetal cortical neurons^a^ ✔ Zebrafish embryos^a^ ✔ Rat adrenal pheochromocytoma PC 12 cells^a^
AO	341	Impairment, learning and memory	✔ Animal experiments

AOP, MIE, KE, and AO as well as their respective event ID were taken from AOP Wiki (https://aopwiki.org/). Evidence checked according to information shown in Tables 2, 3, 4, 5

^a^Indirect evidence

#### AOP-405: organophosphate chemicals induced inhibition of AChE leading to impaired cognitive function

**Fig. 5:**
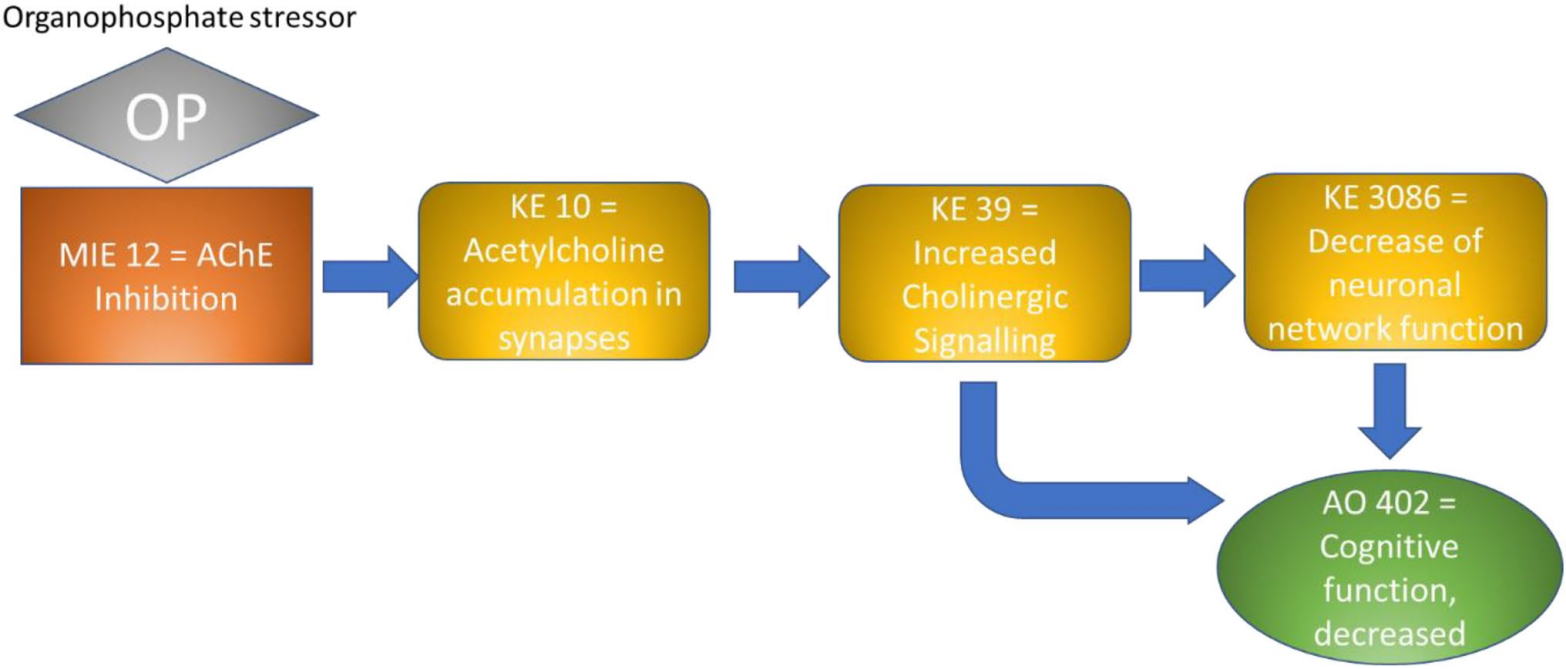
Graphical representation of AOP 405: organophosphate chemicals induced inhibition of AChE leading to impaired cognitive function. Modified from the AOP wiki (https://aopwiki.org/aops/405)

AOP 405 (under development by Amar and Gust 2023) describes how the inhibition of AChE (MIE) by organophosphates (as prototypical stressors) leads to reduced cognitive function (AO 402) (Table 6, Fig. 5). CPF is an organophosphate insecticide with the ability to inhibit AChE, as demonstrated in several NAMs using human (Amaroli et al. 2013; Modefferi et al. 2021) (Table 2) and rodent models (Flaskos et al. 2011; Schuh et al. 2002) (Table 3) as well as zebrafish embryos (Ogungbemi et al. 2020; Rodríguez-Fuentes et al. 2015) (Table 5). It should be noted that other studies report little or no inhibition of AChE by CPF (e.g. Slotkin and Seidler 2012). This may be due to the dose range considered. In fact, CPF is a poor AChE inhibitor, but in the liver it is rapidly converted to its active metabolite CPO (Fig. 1, with an organophosphate backbone), a more potent AChE inhibitor. However, such biotransformation does not always occur with certain NAMs due to the limited or absent metabolic competence of the cellular models used. Despite this limitation, evidence using NAMs support the triggering of MIE 12 (AChE inhibition) by CPF.

After MIE 12, the first KE is ACh accumulation in synapses (KE 10) (Fig. 5, Table 6). We could not find any studies that directly measured ACh in synaptic models. However, this would be expected if AChE, the enzyme involved in stopping nerve transmission by hydrolysing ACh to choline and acetic acid, is strongly inhibited by organophosphates. Several studies, particularly using PC12 cells, have reported cholinergic hyperstimulation (Table 3), which can be seen as indirect evidence of ACh accumulation in the synaptic cleft. This reported cholinergic hyperstimulation is also consistent with the description of the second KE (KE 39) of this AOP (Table 6), the increase in cholinergic signalling. Finally, the third and last KE of this AOP is decrease in neuronal network function (KE 386) (Table 6, Fig. 5), which has been observed in human NSC (Table 2) and in rat cortical neurons (Table 3), often reported together with various disturbances in electrical activity.

Overall, data obtained using NAMs directly support the evidence of, at least, the MIE and one KE (KE 386) of this AOP. Since the reduction in cognitive functions reported after in vivo exposure to CPF is well established (Jett et al. 2001; Roldán-Tapia et al. 2005), the development of NAMs and endpoints supporting the other KEs of the AOP-405 is warranted in the frame of the NGRA.

#### AOP-475: binding of chemicals to ionotropic glutamate receptors leads to impairment of learning and memory via loss of drebrin from dendritic spines of neurons

**Fig. 6:**
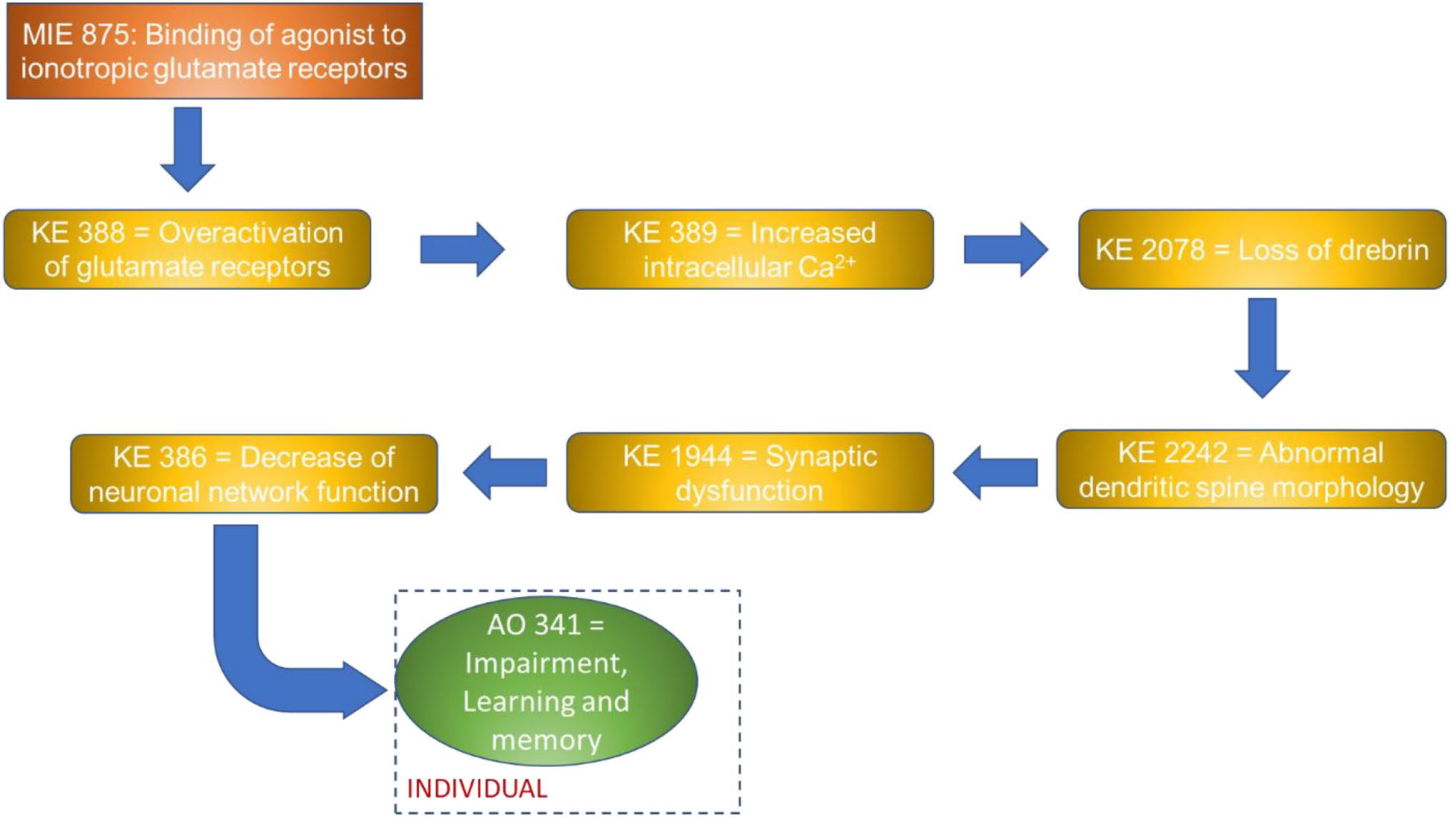
Graphical representation of AOP 475: binding of chemicals to ionotropic glutamate receptors leads to impairment of learning and memory via loss of drebrin from dendritic spines of neurons. Modified from the AOP wiki (https://aopwiki.org/aops/475)

AOP 475 (under development by Sekino 2024, included in the OECD Workplan, Project 1.107) describes how activation of ionotropic glutamate receptors (MIE 875) leads to impairment of learning and memory (AO 341) (Table 6, Fig. 6). In the articles reviewed, we did not find a direct description of MIE 875. Nevertheless, sub-lethal concentrations of CPF potentiated glutamate toxicity in cultured mouse cerebellar granule neurons (Amani et al. 2016) (Table 3), suggesting that CPF is capable to synergise glutamate toxicity by activating glutamate receptors. In addition, the inhibition of glutamate uptake in human foetal astrocytes (Mense et al. 2006) (Table 2) suggests a sustained activation of glutamate receptors, either directly by CPF or indirectly through accumulation of glutamate in the synaptic cleft, thus supporting MIE 875.

The first KE is KE-388, which consists in the overactivation of glutamate receptors (Table 6, Fig. 6). There is indirect evidence for this effect, as the above-mentioned inhibition of glutamate receptors should necessarily induce such overactivation. This is also indirectly supported by the upregulation of the expression of genes encoding glutamate receptors and glutamate transporters in PC12 cells (Slotkin and Seidler 2009a; Slotkin et al. 2010) (Table 3), which could be interpreted as an adaptive response to glutamate receptor overactivation.

As regards the second KE, intracellular calcium overload (KE 389) (Table 6, Fig. 6), we only found that CPF did not affect the depolarisation-induced Ca^2+^ influx in rat cortical cultures (van Melis et al. 2023) (Table 3). However, it should be noted that voltage-gated calcium channels are not the only pathway by which calcium enters the cell, and other pathways remain to be investigated, so it cannot be completely ruled out that this intracellular calcium overload did not occur during CPF exposure.

The next KE in AOP 475 is loss of drebrin (KE 2078) (Table 6, Fig. 6). Drebrin is a cytoskeletal binding protein involved in development (Hotulainen and Hoogenraad 2010). To the best of our knowledge, there are no studies on the effect of CPF on drebrin. However, changes in other cytoskeletal proteins such as a dose-dependent decrease in β-tubulin III in human NSC (Kim et al. 2016), MAP-2 in mouse embryonic stem cells (Visan et al. 2012) and MAP1B in rat C6 glioma cells (Sachana et al. 2008) were observed. Therefore, it seems plausible that when the cytoskeletal ensemble is disrupted, another cytoskeletal protein such as drebrin may not be able to exert its biological function. However, this point remains unclear and deserves further investigation.

Upon loss of drebrin, abnormalities in dendritic spine morphology occurs (KE 2242). Dendritic spines are tiny projections that extend from neuronal dendrites and are responsible for the formation of excitatory synapses in the cortex and hippocampus (Cannizzaro et al. 2019). Dendritic spine morphology has not been studied as such, but the reduction of neurite outgrowth has been described in human NPC (Wu et al. 2017), human NSC (Consiglio et al. 2020; Pistollato et al. 2020), human brain neurospheres (Modafferi et al. 2021), human LUHMES cells (Singh et al. 2018), mouse N2a neuroblastoma cells (Flaskos et al. 2011) and rat adrenal pheochromocytoma PC12 cells (Crumpton et al. 2000a). Therefore, as dendritic spines are part of neuronal dendrites, inhibition of neurite outgrowth must necessarily cause abnormalities in dendritic spine morphology, and therefore, the requirement for KE 2242 appears to be met.

The next KE in this AOP is represented by synaptic dysfunction (KE 1944) (Table 6, Fig. 6). Changes in the number of synapses have been reported in human NSC (Di Consiglio et al. 2020; Pistollato et al. 2020) and in rat cerebellar granule cells (Padhi et al. 2022). This leads to the decrease in neuronal network function (KE 386) (Table 6, Fig. 6), which can be measured by electrophysiological determination of electrical activity using techniques such as patch clamp or microelectrode arrays. In our systematic review, we found several pieces of information warning of changes in electrical activity in the NSC (Di Consiglio et al. 2020) and in foetal rat cortical neurons (Alloisio et al. 2015; Dingemans et al. 2016).

Overall, it can be concluded that there is plausible evidence obtained using NAMs to support the KEs leading to learning and memory impairments reported after in vivo exposure to CPF, triggered by the overstimulation of glutamate receptors with further loss of cytoskeletal proteins and neural network performance. CPF could, therefore, be considered among the prototypical stressors of this AOP.

#### AOP-499: activation of MEK-ERK1/2 leads to deficits in learning and cognition via disrupted neurotransmitter release

**Fig. 7:**
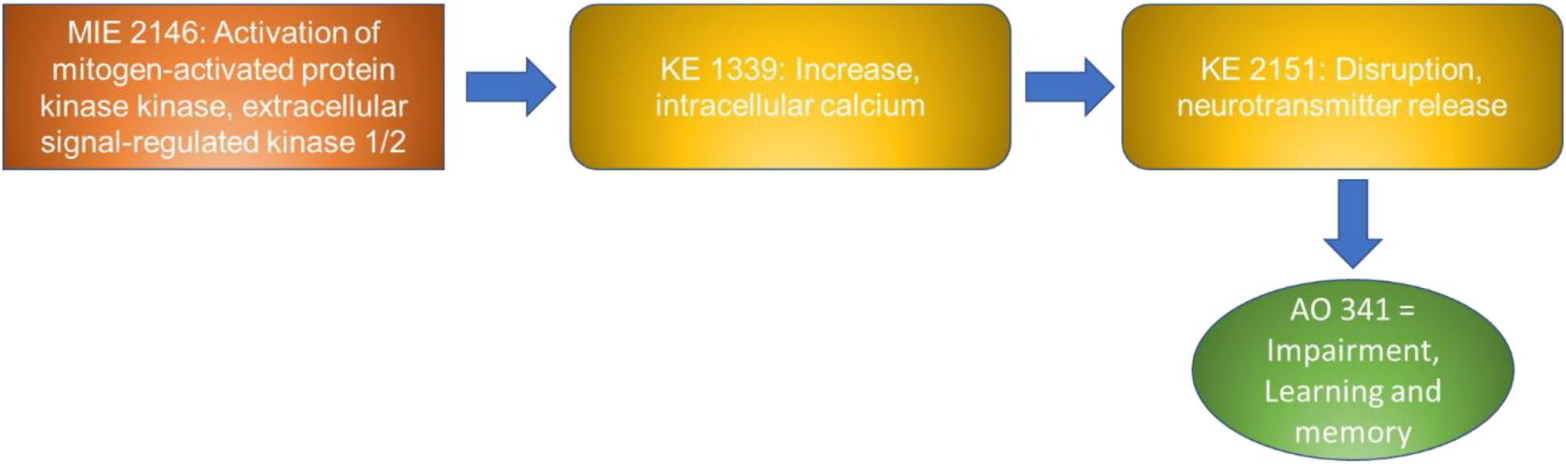
Graphical representation of AOP 499: activation of MEK-ERK1/2 leads to deficits in learning and cognition via disrupted neurotransmitter release. Modified from the AOP wiki (https://aopwiki.org/aops/499)

AOP 499 (under development by von Stackelberg 2024a) starts with the activation of the mitogen-activated protein kinase (MEK), extracellular signal-regulated kinase (ERK) 1/2 (MIE 2146) (Table 6, Fig. 7). In primary human astrocytes, CPF upregulated protein levels of activated ERK 1/2 (Mense et al. 2006). This MIE induces the increase in intracellular calcium (KE 1339), which, although having a different description into the AOP-Wiki, is biologically similar to the one already discussed in relation to AOP 475. The last KE is related to the disruption of neurotransmitter release (KE 2151) (Table 6, Fig. 7). We did not find any direct measurements of neurotransmitter release. Some indirect evidence in rat adrenal pheochromocytoma PC-12 cells suggests that this may occur. Specifically, CPF-induced differentiation towards the dopamine phenotype at the expense of the ACh phenotype (Slotkin and Seidler 2009b). Overall, we have evidence that the MIE of this AOP was triggered by CPF, but more research is needed on several intermediate KEs to consider CPF as a prototypical stressor of this AOP.

#### AOP 500: activation of MEK-ERK1/2 leads to deficits in learning and cognition via ROS and apoptosis

AOP 500 (under development by von Stackelberg 2024b) has the same MIE (2146) and the first KE (1339) as AOP 499. The second KE is related to mitochondrial dysfunction (KE 177). A change in MMP has been described in human NPC following CPF exposure (Mahajan et al. 2019) (Table 2), as well as a reduction in basal respiration and ATP production in N27 rat embryonic mesencephalic dopaminergic neurons (Singh et al. 2018). The conditions for the induction of KE 177 are, therefore, met.

**Fig. 8:**
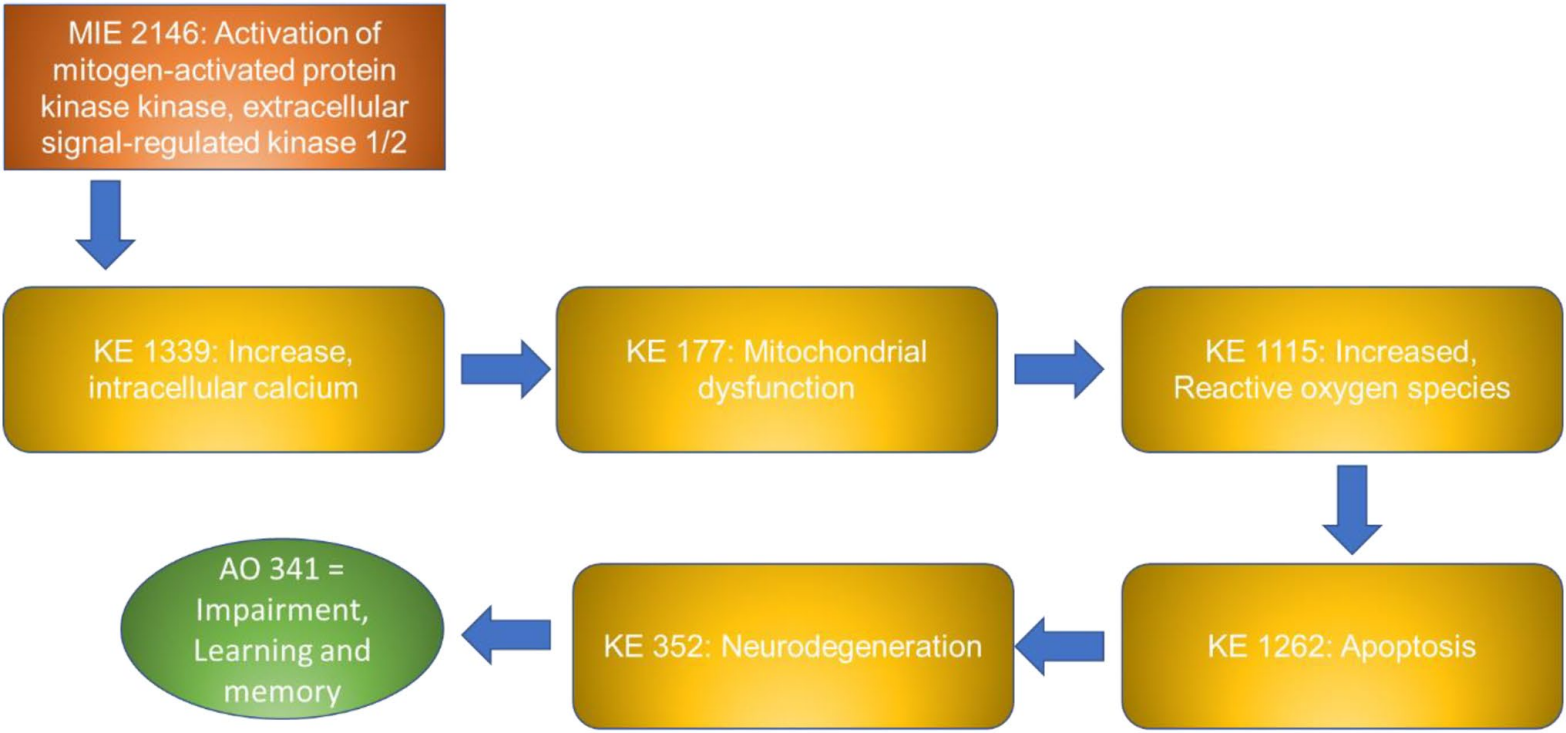
Graphical representation of AOP 500: activation of MEK-ERK1/2 leads to deficits in learning and cognition via ROS and apoptosis. Modified from the AOP wiki (https://aopwiki.org/aops/500)

The next KE, increase in ROS (KE 1115) (Table 6, Fig. 8), is a marker of an increase in oxidative stress which has been described in a large list of studies, including human brain neurospheres (Modafferi et al. 2021), human LUHMES cells (Singh et al. 2018), rat adrenal pheochromocytoma PC-12 cells (Crumpton et al. 2000a), N27 rat embryonic mesencephalic dopaminergic neural cells (Singh et al. 2018), mouse cultured cerebellar granule neurons (Amani et al. 2016) and rat C6 glioma cells (García et al. 2001). This leads to apoptosis (KE 1262), which has been demonstrated in human NPC (Mahajan et al. 2019), human LUHMES cells (Singh et al. 2018), rat foetal cortical neurons (Caughlan et al. 2004), and zebrafish embryos (Yu et al. 2015).

Finally, neurodegeneration (KE 352) is the last KE of the AOP 500 (Table 6, Fig. 8). Histopathological evidence of neurodegeneration is not easily obtained using NAMs. However, according to the description in the AOP-Wiki, neurodegeneration can be investigated when some of the following events occurs: loss of mitochondrial function, changes in the activity of neuronal networks and decreased levels of neurotrophic factors. The first two events have already been discussed above for other KEs. The last one has been demonstrated in rat adrenal pheochromocytoma PC-12 cells with, among others, impairment of Ngf or neurotrophic factor or neurotrophic tyrosine kinase receptor (Slotkin et al. 2008).

Overall, it can be concluded that there is plausible evidence obtained using NAMs to support CPF as prototypical stressor of this AOP.

#### Possible synergism between different mode of actions: an AOP network proposal

**Fig. 9:**
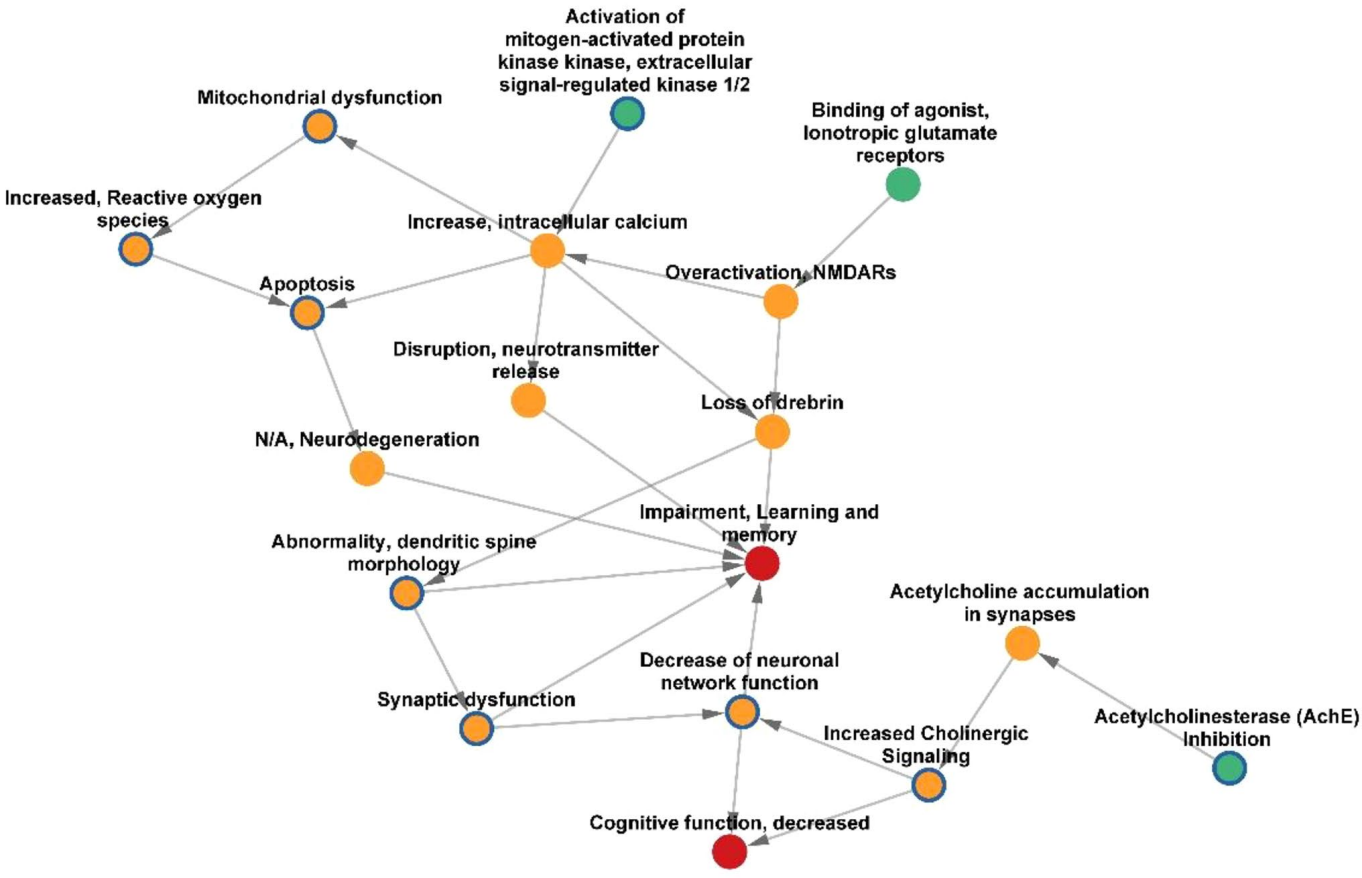
Network view of the relationships between the AOPs that are proposed to intervene in the mode of action of the developmental toxicity induced by CPF. MIEs are in green, KEs are in orange and AOs are in red. Events for which we found evidence in this review have a blue border. For mechanistic reasons, we merged the KE 1339 (Increase, intracellular calcium) and KE 389 (Increase, intracellular calcium overload)

It is noteworthy that the possibility of synergism between these four described AOPs cannot be ruled out, since three of them share a KE consisting in an increase of intracellular calcium (AOP 475, AOP 499 and AOP 500) and two others (AOP 405 and AOP 475) share the KE related to the decrease of neuronal network function (Fig. 9). Overall, evidence collected in this review based on NAMs, directly support the developmental plausibility of some KEs with CPF as stressor, for which plausibility exists also in adults. For other KEs (e.g. activation of glutamate receptors, disruption of neurotransmitter release or ACh accumulation in synapses) plausibility of CPF effects during development could be postulated by NAMs data, although more research is needed to provide direct evidence. Finally, AOs are strongly supported by animal rodent studies; available behavioural effects on zebrafish embryos following exposure to CPF are still limited, but these are promising models to investigate also these outcomes in detail.

## Conclusions

There is a good consensus among experts that a single NAM would never be able to elucidate a mode of action, especially in biological processes as complex as development. Such multifaceted problems should be addressed by IATA to gather all the available evidence on a particular question. In this work, we have compiled and integrated all available information obtained through NAMs about the developmental toxicity of CPF. We conclude that, overall, the observed adverse effects are relevant to humans, although not all have been fully confirmed in epidemiological studies. Moreover, building upon available AOPs, it is biologically plausible that CPF alter development through a mixed mode of action based on persistent inhibition of AChE (AOP 405), overstimulation of glutamate receptors causing loss of drebrin from dendritic spine of neurons (AOP 475) and activation of ERK 1/2 leading to neurotransmitter release disruption or oxidative stress and apoptosis (AOP 500). All these AOPs end with impairment of cognition or learning and memory as AOs, which has been demonstrated in a number of in vivo studies to be associated with CPF exposure. Thus, increasing the plausibility of the mechanism.

This risk of human developmental toxicity is outside the scope of this work and is difficult to be addressed solely on the basis of NAMs without the support of toxicokinetic information, which we have not included in our assessment. Traditionally, the risk of developmental toxicity in humans has been considered to be low, as the acute maternal cholinergic effect may prevent such effects. However, this manuscript shows that the mode of action is not only through AChE inhibition but also through another AChE-independent mechanism at doses below those altering AChE activity, or that do not involve AChE inhibition. Therefore, the risk of CPF developmental toxicity in humans remains to be elucidated and clarified and clearly merits future research efforts. In addition, in almost all the NAMs here described, the endpoints were related to developmental neurotoxicity with only very limited examples in zebrafish models assessing CPF endocrine disrupting effects. Efforts in investigating such outcomes by NAMs should be, therefore, increased.

## Supplementary Information

Below is the link to the electronic supplementary material.

Supplementary file1 (XLSX 42 KB)

## Abbreviations

5HT: Serotonin
AC: Adenylyl cyclase
ACh: Acetylcholine
AChE: Acetylcholinesterase
AhR: Aryl hydrocarbon receptor
AO: Adverse outcome
AOPs: Adverse outcome pathways
BDNF: Brain-derived neurotrophic factor
CNS: Central nervous system
CPF: Chlorpyrifos (O, O′-dithyl-O-3,5,6-trichloro-2-pyrydyl phosphorothionate)
CPO: Chlorpyrifos-oxon (O, O′-dithyl-O-3,5,6-trichloro-2-pyrydyl phosphate)
DIV: Days in vitro
DMH3Lys4: Di-methylated lysine 4 of histone H3
dpf: Days post-fertilisation
dph: Days post hatching
ED: Embryonic day
EFSA: European Food Safety Agency
ERK: Extracellular signal-regulated kinase
hpf: H post-fertilisation
HUCB-NSC: Human umbilical cord blood-derived neural stem cell
IATA: Integrated Approaches to Testing and Assessment
IC_50_: Concentration that reduces cell viability, enzyme inhibition or protein or gene expression by 50%
iPSC: Induced pluripotent stem cells
IR: Immunoreactivity
KE: Key event
MAP-2: Microtubule-associated protein 2
MEK: Mitogen-activated protein kinase
MIE: Molecular initiating event
MMP: Matrix metalloproteinase
NAMs: New Approach Methods
NGF: Nerve growth factor
NPC: Neuronal progenitor cells
NSC: Neuronal stem cells
